# AKAP200 promotes Notch stability by protecting it from Cbl/lysosome-mediated degradation in *Drosophila melanogaster*

**DOI:** 10.1371/journal.pgen.1007153

**Published:** 2018-01-08

**Authors:** Neeta Bala Tannan, Giovanna Collu, Ashley C. Humphries, Ekatherina Serysheva, Ursula Weber, Marek Mlodzik

**Affiliations:** 1 Dept. of Cell, Developmental, and Regenerative Biology, Icahn School of Medicine at Mount Sinai, New York, New York, United States of America; 2 Graduate School of Biomedical Sciences, Icahn School of Medicine at Mount Sinai, New York, New York, United States of America; 3 Tisch Cancer Institute, Icahn School of Medicine at Mount Sinai, New York, New York, United States of America; Harvard Medical School, Howard Hughes Medical Institute, UNITED STATES

## Abstract

AKAP200 is a *Drosophila melanogaster* member of the “A Kinase Associated Protein” family of scaffolding proteins, known for their role in the spatial and temporal regulation of Protein Kinase A (PKA) in multiple signaling contexts. Here, we demonstrate an unexpected function of AKAP200 in promoting Notch protein stability. In *Drosophila*, *AKAP200* loss-of-function (LOF) mutants show phenotypes that resemble *Notch* LOF defects, including eye patterning and sensory organ specification defects. Through genetic interactions, we demonstrate that *AKAP200* interacts positively with *Notch* in both the eye and the thorax. We further show that AKAP200 is part of a physical complex with Notch. Biochemical studies reveal that AKAP200 stabilizes endogenous Notch protein, and that it limits ubiquitination of Notch. Specifically, our genetic and biochemical evidence indicates that AKAP200 protects Notch from the E3-ubiquitin ligase Cbl, which targets Notch to the lysosomal pathway. Indeed, we demonstrate that the effect of AKAP200 on Notch levels depends on the lysosome. Interestingly, this function of AKAP200 is fully independent of its role in PKA signaling and independent of its ability to bind PKA. Taken together, our data indicate that AKAP200 is a novel tissue specific posttranslational regulator of Notch, maintaining high Notch protein levels and thus promoting Notch signaling.

## Author summary

AKAP200 belongs to a family of scaffolding proteins best known for their regulation of PKA localization. In this study, we have identified a novel role of AKAP200 in Notch protein stability and signaling. In *Drosophila melanogaster*, AKAP200’s loss and gain-of-function (LOF/GOF) phenotypes are characteristic of Notch signaling defects. Furthermore, we demonstrated genetic interactions between *AKAP200* and *Notch*. Consistent with this, AKAP200 stabilizes the endogenous Notch protein and limits its ubiquitination. AKAP200 exerts its effects on Notch by antagonizing Cbl-mediated ubiquitination and thus lysosome targeting of Notch. Based on these data, we postulate a novel PKA independent mechanism of AKAP200 to achieve optimal Notch protein levels, with AKAP200 preventing Cbl-mediated lysosomal degradation of Notch.

## Introduction

Signaling pathways are critically involved throughout embryonic development, as well as adult tissue function and homeostasis. Many of these pathways are highly conserved from invertebrates to humans, and were first discovered in *Drosophila melanogaster*, making it an ideal model system for identification and analysis of new pathway components. Notch signaling, is one such pathway, and is required for fundamental developmental processes including polarity, cell fate specification, tissue growth, stem cell maintenance, and organ patterning [1–5]. Moreover, misregulation of Notch signaling underlies several human diseases including various cancers highlighting the importance of Notch pathway regulation [1, 3, 6–8].

In *Drosophila*, Notch signaling is activated by the interaction of the ligands Delta and Serrate with the extracellular domains of the Notch receptor [9]. Ligand binding activates extracellular cleavage of Notch by ADAM/TACE metalloproteases [10], followed by γ-secretase mediated cleavage [11], which releases the Notch intracellular domain (NICD) [12]. The NICD, which is the signal transducing end of the protein, enters the nucleus, forms a complex with the transcription factor Suppressor of Hairless [Su(H)]/CSL and activates target genes [5, 13–15]. The same fundamental elements/mechanisms of the pathway are conserved in mammals [16, 17]. Notch signaling is tempered by endocytosis of the receptor and degradation of NICD and these processes are essential to avoid hyperactivity [18, 19]. Several studies have demonstrated proteasomal degradation of Notch. For example, dominant negative mutations of proteasomal subunits enhance Notch signaling in *Drosophila* [20]. Initial evidence for lysosomal degradation of Notch came from a study in skeletal myoblasts, the C2C12 cell line, where a role was demonstrated for c-Cbl (Casitas B-lineage lymphoma, a proto-oncogene and E3 ubiquitin ligase) in mono-ubiquitinating the endogenous transmembrane Notch1 and targeting it for lysosomal degradation [21]. Suppressor of Deltex [Su(dx)]/Itch (*Drosophila/*mouse) and Sel10 have been shown to decrease Notch signaling in this context [22–25].

Mutations in Notch affect many developmental decisions in various *Drosophila* tissues [26, 27]. For example, Notch signaling instructs specification of the eye field and initiation of eye development, as well as controlling growth and cell fate [28–31]. The interplay between Notch and Frizzled (Fz)/Planar Cell Polarity (PCP) signaling is critical for induction of specific photoreceptor (PR) subtypes [29, 30, 32–34]. In the developing eye disc, there is a Frizzled/PCP activity gradient that is highest at the dorso-ventral midline, termed the equator, and lowest at each pole [30, 35, 36]. Within each developing PR cluster, there are pairs of cells that are initially equivalent that then develop into photoreceptor 3 and 4 (R3 and R4). Within each pair, the cell that is closest to the midline adopts the R3 fate and upregulates the Notch ligand *Delta*, and *neuralized* and signals via Notch to its polar neighbor to adopt the R4 fate [30, 36–38].

In a screen for novel regulators of PCP signaling in the *Drosophila* eye, we identified a scaffolding protein, A Kinase Anchoring Protein 200 (AKAP200) [39]. AKAPs are a family of proteins responsible for the subcellular compartmentalization of Protein Kinase A (PKA), which facilitate the spatial and temporal regulation of signaling [40–42]. Despite being structurally and sequentially diverse, the AKAP family of proteins show functional conservation amongst species [43]. AKAP200 is one member of the AKAP family of proteins and is expressed throughout all stages of *Drosophila* development. Alternative splicing produces two isoforms of AKAP200—the full length AKAP200-Long (AKAP200-L), and the short isoform, AKAP200-Short (AKAP200-S). AKAP200-S lacks the PKA-interaction domain and may therefore be limited to PKA- independent functions [44, 45].

Here we provide evidence that AKAP200 is required for the regulation of Notch protein levels, via the lysosomal degradation pathway. AKAP200’s loss and gain-of-function (LOF/GOF) phenotypes are characteristic of misregulation of the Notch signaling pathway. AKAP200 LOF mutants display defects in cell fate specification manifested as loss of PRs in the eye and extra sensory bristles in the thorax, while AKAP200 overexpression causes wing vein defects and tissue overgrowth in the wing. Genetic interaction studies revealed that AKAP200 acts as a positive regulator of Notch signaling, as loss of AKAP200 suppresses Notch overexpression phenotypes in the eye and thorax. Consistent with this, we observe a decrease in overall Notch protein levels and increased ubiquitination in the *AKAP200* mutant relative to *wild type (WT)*. Importantly, AKAP200’s effects on Notch are independent of PKA. However, we find that the suppression of Notch hyperactivity in *AKAP200* mutant tissues is instead dependent on the E3 ubiquitin ligase Cbl and the lysosomal degradation pathway. Based on these data, we postulate a novel mechanism for the regulation of Notch levels, with AKAP200 preventing Cbl-mediated lysosomal degradation of Notch.

## Results

### *AKAP200* mutants display Notch like phenotypes

To identify novel genes involved in PCP-mediated photoreceptor specification, we performed a genetic screen for dominant modifiers of a gain-of-function (GOF) of the core PCP factors *dgo* and *pk* [39]. Overlapping deficiencies narrowed down a region on chromosome 2L that enhanced the *dgo* GOF PCP eye phenotypes (S1A Fig). Further analysis using RNA interference (IR) against specific genes in this interval revealed that A Kinase Anchoring Protein 200 (AKAP200) reproduced the interaction, implicating it as the gene responsible (S1A and S1B Fig).

**Fig 1:**
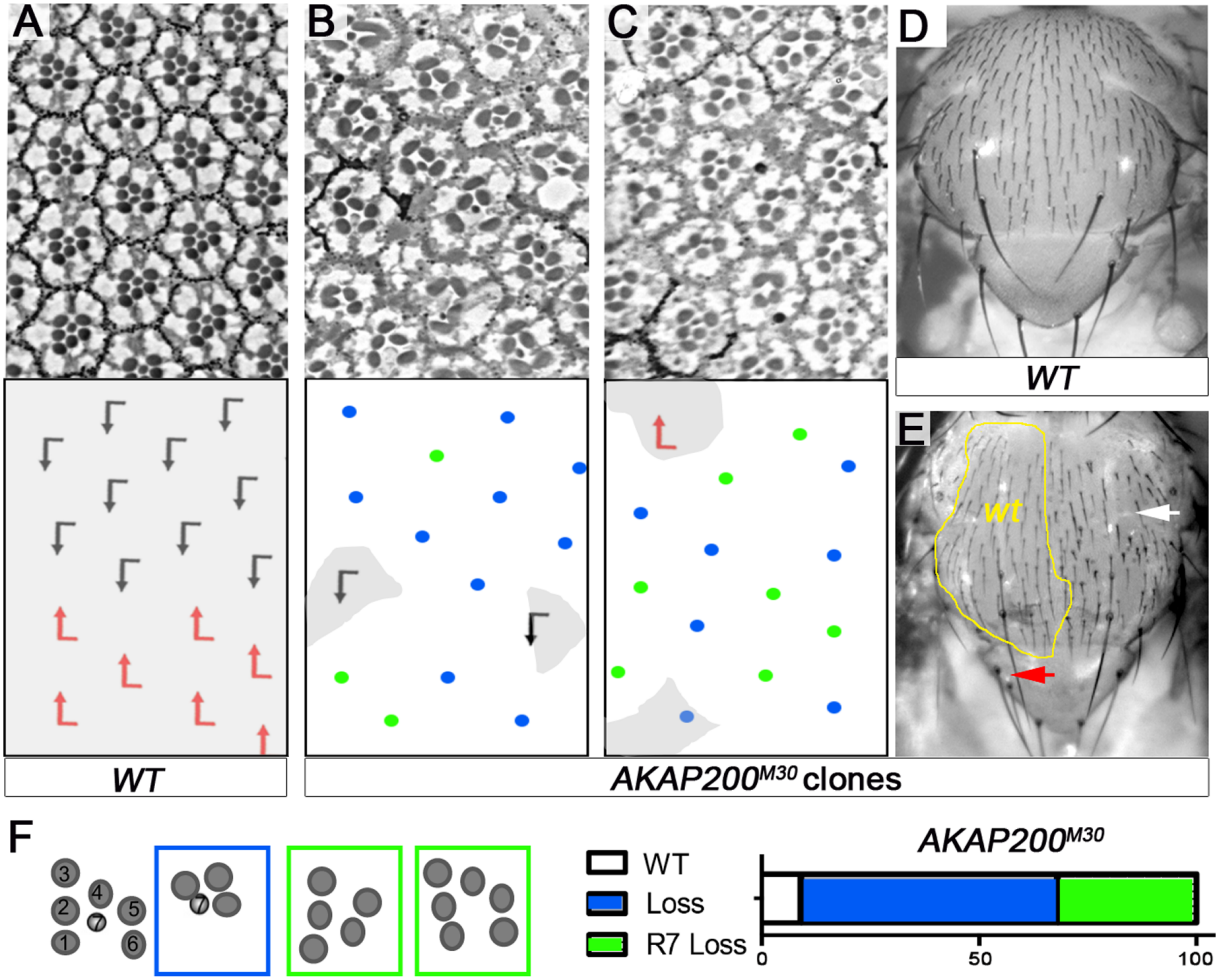
*AKAP200* mutants show phenotypes that resemble *Notch* LOF in adult Drosophila eye and thorax. (A-C) Tangential adult eye sections with anterior left and dorsal up. (A) Example of *wild type* (*WT)* eye tissue identified by presence of pigment granules (shaded grey, also in B and C, in schematics). (B-C) *AKAP200* mutant tissue, lacking pigment (clonal marker used was *w*^*+*^, small *wt* areas shaded grey). (B) Dorsal and (C) ventral eye regions, with *AKAP200*^*M30*^ tissue displaying frequent loss of photoreceptors (schematized in lower panels); see panel (F) for key and quantification. (D-E) Thorax of indicated genotypes, anterior is up. (D) *WT* control showing normal sensory bristle pattern. (E) *AKAP200* mutant clones (marked by absence of *y*^*+*^, some *wt* patches outlined with yellow line) display defects in SOP specification, resembling Notch signaling defects, as evident by bald spots (white arrow) and supernumerary scutellar bristles (example highlighted by red arrow). (F) Schematic of different ommatidial phenotypes observed in (B-C). Blue box/dots depict loss of one or several outer photoreceptors and green boxes/dots depict loss of R7, with or without simultaneous loss of outer photoreceptors. Graph: quantification of distribution of phenotypes in *AKAP200*^*M30*^ (n = 666 from 8 eyes).

To investigate AKAP200 functions, we first generated mutant alleles by excising the coding sequence of *AKAP200* using flanking *piggyBac/FRT* insertions (S1C Fig). This led to the isolation of two null alleles, *AKAP200*^*M30*^ and *AKAP200*^*M24*^ that were confirmed by PCR characterization (S1D Fig; see [46] for method). These were lethal homozygous, or transheterozygous over a deficiency chromosome, with rare escapers (S1G–S1J Fig). To analyze the loss-of-function (LOF) phenotypes we generated mutant clones via the Flp-FRT system [47]. In the eye, *AKAP200*^*M30*^ mutant clones displayed loss of or misspecification of photoreceptors (PRs) (Fig 1B, 1C and 1F). These phenotypes were also consistently seen in escapers from different *AKAP200* transheterozygous LOF genetic backgrounds and also mimicked the phenotypes seen with *AKAP200-IR* knock-downs (S1G and S1H Fig; these were often quantitatively weaker than the null clones). A frequent defect was loss of R7 in mutant ommatidia (Fig 1B, 1C and 1F), with R7 specification requiring both Notch and RTK (Sev and Egfr) signaling. To confirm this we analyzed developing eye discs with molecular markers (Elav to stain all R-cells and Pros labeling R7), which revealed partial photoreceptor specific loss of Pros staining in mutant eye discs (S1I and S1I’ Fig). Together with the identification in the PCP screen, the observed eye phenotype was suggestive of a possible link to Notch function, with similarities to aspects of *Notch* LOF phenotypes [28, 48–50]. Also as an interplay between Notch and Fz/PCP signaling is critical for R3/R4 specification and PCP patterning in the eye, regulators of either pathway were expected to be and were identified in the screen [39].

The *AKAP200* LOF thorax phenotype [generated using *ubx-Flp* inducing clones in all imaginal discs at early larval stages [51]] revealed supernumerary scutellar bristles, and loss of or mispositioned microchaetae (Fig 1E, red arrow marks supernumerary bristles and white arrow a bald patch representing loss of bristles, compare to wild-type area with evenly spaced and regular positioning of microchaetae bristles). Strikingly, the supernumerary scutellar bristles (macrochaetae; Fig 1E, red arrow) are a hallmark *Notch*^-^/+ haploinsufficiency phenotype, and this resembled *Notch* signaling defects. *Notch* is required at multiple steps in the process specifying SOPs (sensory organ precursors) and the different cell types originating from them, including the positioning and spacing of SOPs and asymmetric activation of Notch during their multiple asymmetric divisions. Based on at what time point in this process Notch signaling is disrupted, a variety of phenotypes can be expected [52–58]. When analyzed in pupal thorax clones, relative to neighboring *wild type* (*WT*), *AKAP200* mutant tissue displayed loss of SOPs and SOP mispositioning (S1E and S1F Fig), as well as rare SOP lineage defects (Fig 1E and S1F and S1F’ Fig, arrowheads).

In the wing, *AKAP200* mutant wings appeared blistered as did mutant clones, but we did not observe defects to the margin (S1J Fig). Interestingly, the blistered wing phenotype suggested a PKA related function of AKAP200 [59, 60], whereas the eye and thorax phenotypes resembled a subset of Notch LOF defects with no obvious link to PKA signaling. Taken together, these phenotypic defects suggest that *AKAP200* might affect Notch signaling in the eye and thorax, whereas it seems to act ‘canonically’ via PKA in wings.

### AKAP200 promotes Notch signaling

The *AKAP200* mutant phenotypes resembled that of *Notch* LOF in eyes and the thorax, suggesting a novel function for AKAP200. To gain further insight into its potential involvement in the Notch pathway, we tested for genetic interactions between *AKAP200* and *Notch*-associated genotypes (LOF and GOF) in the eye and thorax.

**Fig 2:**
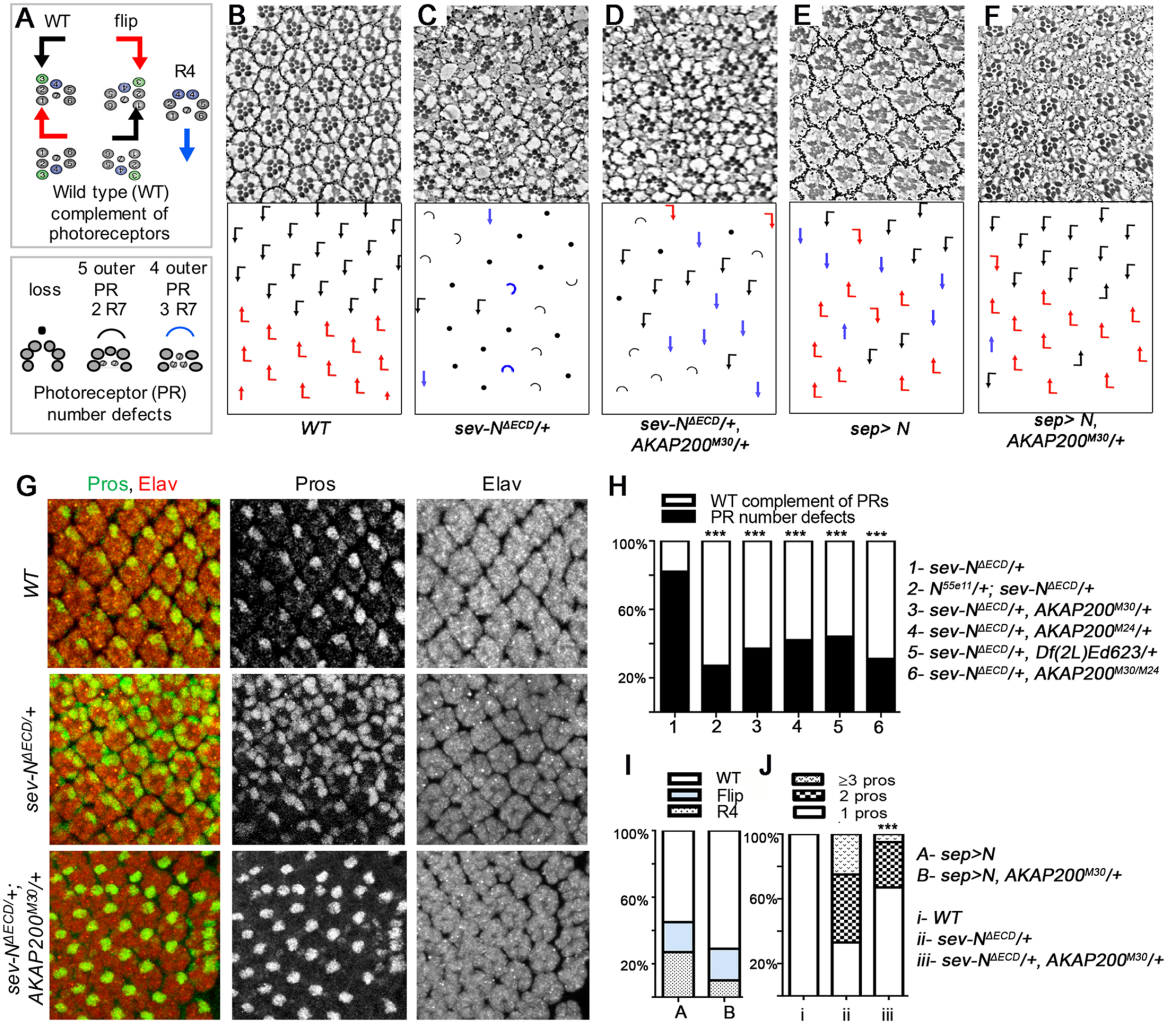
AKAP200 promotes Notch signaling. (A) Schematic of eye phenotypes. Ommatidia with 6 outer PRs and 1 R7 are classified as WT complement of PRs: WT PR clusters are represented by flagged-arrows, black and red respectively for dorsal and ventral chiral forms, intermixing of these indicates flips. Blue arrows represent symmetrical clusters with two R4s. Loss of any PR is indicated with a black dot, black and blue semi-circles represent 5 outer PRs and 2 R7s, and 4 outer PRs and 3 R7s, respectively. Any ommatidia lacking a PR (outer or R7) are classified as PR number defects in quantifications, unless otherwise indicated. (B-F) Tangential adult eye sections of indicated genotypes; anterior is left and dorsal up in all panels with schematics in lower panels. (B) *WT* control, note regular arrangement of chiral forms in a mirror symmetric manner across the equator. (C-D) The heterozygous *AKAP200* null mutant markedly suppresses the phenotype of the membrane tethered activated *sev-N*^*ΔECD*^, which is ligand independent, quantified in (H). (E-F) *sep-Gal4*, *UAS-N* flies raised at 18°C show chirality flips and R4/R4 symmetrical clusters (E). Note that *AKAP200*^*M30*^*/+* suppresses *sep-Gal4*, *UAS-N* induced R4/R4 symmetrical clusters. Quantified in (I). (G) Confocal images of third instar eye discs of indicated genotypes stained for neuronal marker Elav (red, labeling all PR cells) and Pros (green, labeling R7). One R7/ommatidium is observed in *WT* (top row). Activation of Notch signaling by *sev-N*^*ΔECD*^ increases R7s/ommatidium (middle row), which is suppressed with simultaneous reduction of *AKAP200* (bottom row; quantified in J). (H) *AKAP200* mutants suppress Notch PR number defects (including loss, transformation of outer PRs to 2 or 3 R7s). Quantification of genotypes of *sev-N*^*ΔECD*^ related to (C-D) as indicated. *sev-N*^*ΔECD*^ causes PR number defects which were reduced in *sev-N*^*ΔECD*^*/+*, *AKAP200*^*M30*^*/+* and reproducible in heterozygous and homozygous *AKAP200* null mutants (*M30* and *M24*) and deficiency. ***p<0.0001 by chi square test (against *sev-N*^*ΔECD*^*/+*), n = 320–569 from 3–4 independent eyes. (I) Quantification of genetic interactions in (E-F). *sep>N* caused R4 symmetrical clusters (27% ± 1%). In *sep>N*, *AKAP200*^*M30*^*/+* these were reduced to (9% ± 3%) (***p<0.0001 by chi square test, from 3–4 independent eyes). (J) Quantification of (G), n = 163–234 ommatidia from 7 independent eyes; ***p<0.0001 by chi square test.

The *Drosophila* eye is a compound eye with a mirror symmetric organization of ommatidia across the dorso-ventral midline [61] (Fig 2A and 2B). Perturbation of Notch signaling during ommatidial assembly can lead to a variety of phenotypes [28, 31, 33, 50]. These can be classified into ommatidia with the WT complement of photoreceptors, PRs (6 outer photoreceptors and single R7/R8) and include altered ommatidial orientation, flips, or clusters with a symmetrical appearance; and ommatidia with PR number defects (for example loss or gain of R7; Fig 2A).

To probe the relationship of *AKAP200* and the Notch pathway, we asked whether *AKAP200* mutants influenced Notch signaling in the eye. N^ΔECD^, a membrane-tethered deletion of the extracellular domain that renders Notch active in a ligand-independent manner [62], was expressed under the control of the *sevenless (sev)* promoter [which is initially expressed in R3-R4, and later in R1,6 and 7 and cone cells [63]]. *sev-N*^*ΔECD*^ causes the formation of R4 symmetrical clusters and chirality flips besides frequent defects in PR number and supernumerary R7s (Fig 2C and 2H). Reducing the copy number of *AKAP200* in the *sev-N*^*ΔECD*^ background markedly reduced these defects. Whereas PR cell loss was reduced, a proportional increase was seen in less severe *Notch* GOF phenotypes, including R4 symmetrical clusters and chirality flips (Fig 2D and 2H). The suppression of Notch overactivation by *AKAP200*^*M30*^ was comparable to reducing Notch protein levels, e.g. *Notch*^-^*/+* (Fig 2H). These interactions were reproducible with all *AKAP200* alleles and deficiencies tested, and was strongest upon homozygous removal of *AKAP200* (*AKAP200*^*M30/M24*^*)* in rare escapers (Fig 2H), altogether suggesting that AKAP200 promotes Notch signaling. To confirm this assessment, we tested additional phenotypes associated with the activated Notch pathway. A milder Notch GOF background, using a weaker *sev-Gal4* driver (*sev*-enhancer with *sev* promoter: “*sep>*”) resulted in chirality defects with flips and R4 symmetrical clusters (Fig 2E and 2I). Upon removing one copy of *AKAP200* in this background, the number of R4 symmetrical clusters was markedly reduced (Fig 2F and 2I), again consistent with the notion that AKAP200 promotes Notch signaling.

To confirm these interactions, we next analyzed larval eye discs for the expression of Prospero (Pros), a molecular marker for R7 (and also later in cones cells) (Fig 2G; Elav, was used as a pan-neuronal marker to stain all PRs). In contrast to *WT*, where each ommatidium has a single Pros positive R7, in *sev-N*^*ΔECD*^ most ommatidia had 2 or 3 Pros positive R7s. Removing a copy of *AKAP200* suppressed this phenotype, with an appearance closer to *WT* (Fig 2G and 2J). These observations are consistent with the interactions above and correlate with phenotypes seen in adult eyes (Fig 2C, 2D and 2H). We next performed the equivalent experiment using a Notch signaling reporter, *mδ0*.*5-lacZ*, a 500 bp fragment of the *E(spl)mδ* promoter [37], which serves as a molecular readout of Notch signaling specifically in R4 (it is initially expressed at low levels in the R3/R4 pair, and following Notch activation it is upregulated in R4). In *WT*, each ommatidium displayed a single *mδ-lacZ* positive cell, whereas, in contrast, *sev-N*^*ΔECD*^ eye discs displayed generally 2 *mδ-lacZ* positive cells per ommatidium. Consistent with the effects in adult eyes, removing one copy of *AKAP200* in the *sev-N*^*ΔECD*^ background suppressed this phenotype, with most ommatidia displaying only one *mδ-lacZ* positive cell (S2K and S2L Fig).

In order to solidify the notion that AKAP200’s eye phenotypes are directly related to Notch signaling or whether other pathways are involved, we also tested for a potential AKAP200 involvement with Egfr signaling, which has similar phenotypes [64, 65]. To do this, we performed genetic interactions with Egfr GOF by using the *Egfr*^*Elp1*^*/+* allele (S2M Fig) and saw no significant difference in its PR number defect phenotype when one copy of *AKAP200* was removed. Furthermore, we performed this interaction in the wing. Again, *AKAP200*^*M30*^*/+* (S2O Fig) was unable to modify the ectopic vein phenotypes of *Egfr*^*Elp1*^*/+* (S2N Fig).

We next wished to confirm the link between *Notch* and *AKAP200* in the thorax, as *AKAP200* LOF displayed supernumerary scutellar macrochaetae defects (Fig 1E, red arrow), which highly resemble the Notch haploinsufficiency phenotype. Strikingly, an increase in *Notch* copy number (3 copies) suppressed the macrochaetae defects of *AKAP200* LOF mutants (S3N and S3O Fig). In contrast, removing one copy of *AKAP200* enhanced the *Notch* haploinsufficiency (*N*^*55e11*^*/+*) thorax phenotype (quantified in S2F Fig, depicted in S2G–S2J Fig). These data confirmed a positive requirement for AKAP200 in Notch signaling and suggested that AKAP200 might affect Notch levels (see below). Since the *N* null alleles do not display haploinsufficient phenotypic defects in the eye, analogous eye experiments could not be tested.

To further corroborate the link between AKAP200 and Notch signaling, we tested Notch GOF genotypes in the thorax. Overactivation of Notch signaling in the thorax can be achieved via the haploinsufficient *Hairless* (*H*) mutant. H is a nuclear antagonist of Notch signaling and represses Notch target genes by assembling a transcriptional repressor complex [66]. H is involved in neuronal fate specification, and the mutant thorax phenotype reflects overactivated Notch signaling during SOP specification, resulting in reduction of bristles (quantified in S2A Fig, depicted in S2B and S2C Fig). Su(H) is the DNA-binding transcription factor that is directly bound by NICD. H antagonizes Su(H)’s ability to bind to NICD and thereby activate transcription [67], consistent with known interactions between *Su(H)* and *H/+* (quantified in S2A Fig, depicted in S2D Fig). Similarly, *N*^*55e11*^*/+* suppressed *H/+* albeit to a lesser degree (S2A Fig). Supporting the interactions in the eye, *AKAP200*^*M30*^ and *AKAP200*^*M24*^, as well as the AKAP200 deficiency suppressed the *H/+* phenotype (S2A Fig, depicted in S2E Fig). Taken together, the data from the eye and thorax are consistent with *AKAP200* promoting Notch signaling activity.

### AKAP200 promotes Notch signaling in a PKA-independent manner

**Fig 3:**
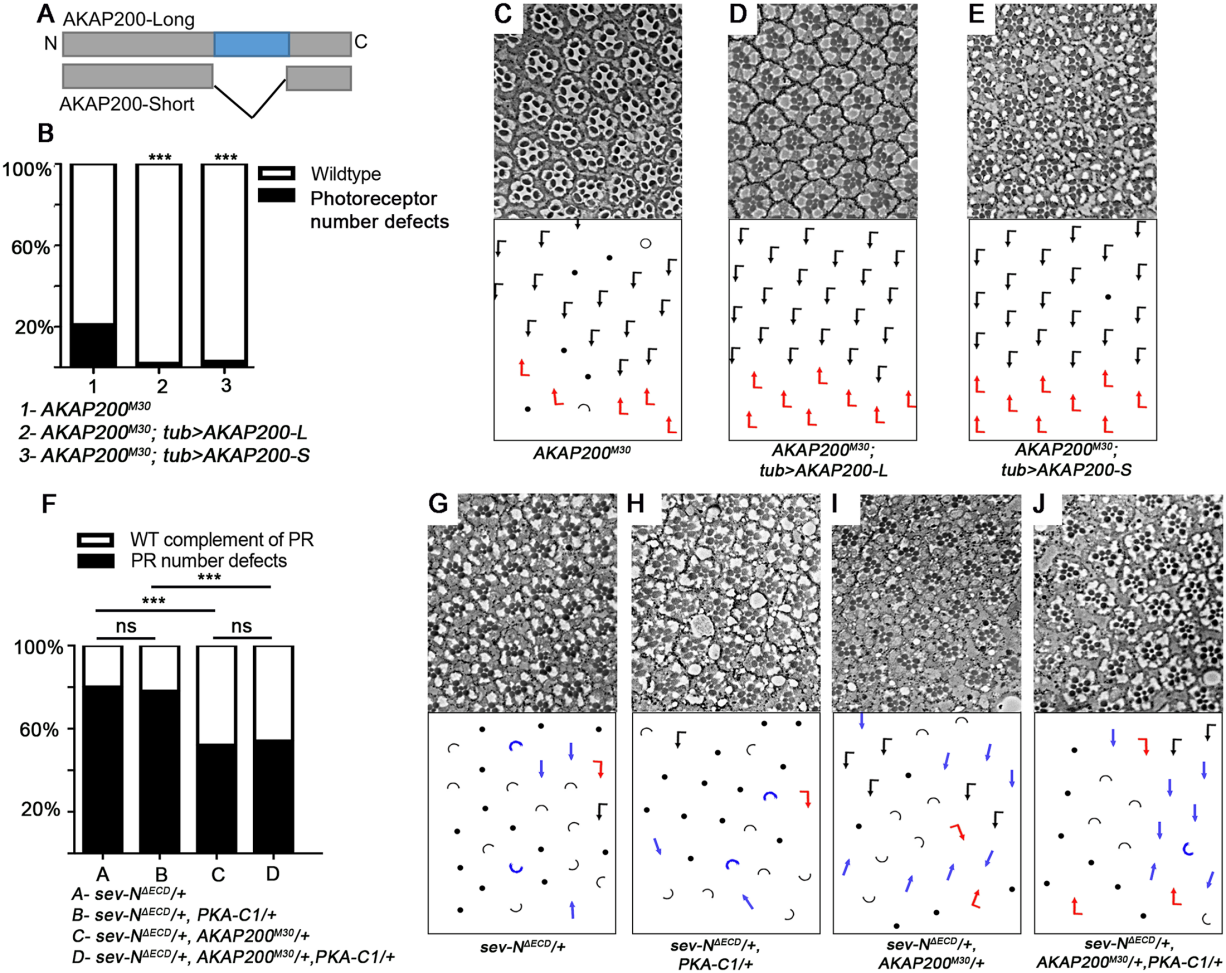
AKAP200 promotes Notch signaling in a PKA independent manner. (A) *AKAP200* has 2 splice variants. AKAP200-L, which can interact with the regulatory subunits of PKA via a tethering site coded for by exon 5 (blue). This exon is spliced out in AKAP200-S, eliminating its ability to interact with PKA. (B-E) Both AKAP200 isoforms can rescue PR number defects in the eye and lethality. Note in schematic of eye phenotypes, loss is indicated by a solid black dot and loss specifically of R7 is indicated by a hollow dot (B) Quantification of genotypes shown in (C-E) ***p<0.0001 by chi square test (against *AKAP200*^*M30*^, n = 514–726, from 3 independent eyes). (C-E) Tangential adult eye sections of indicated genotypes (C) Homozygous *AKAP200*^*M30*^ escaper displays PR number defects. Expression of *AKAP200-L* (D) and *-S* (E) via *tubulin-Gal4* rescues the *AKAP200* phenotype, suggesting that this phenotype is PKA independent. (F-J) PKA-independent effects of AKAP200 on N signaling. (F) Quantification of genotypes shown in (G-J), ***p<0.0001, by chi square test (n = 320–573 from 3–4 independent eyes). (G-J) Tangential adult eye sections of indicated genotypes. PR number defects caused by *sev-N*^*ΔECD*^ (G) is not modified by *PKA*^*-/+*^ (H), but is suppressed by *AKAP200* mutant (I), or both together (J). There is no statistical difference in the effect on *sev-N*^*ΔECD*^ of removing either one copy of A*KAP200* alone or together with *PKA*, suggesting that PKA may not be required for AKAP200’s effect on Notch signaling.

Since AKAP200 is known to interact with and confine the cellular localization of PKA, we next determined whether AKAP200’s effects on Notch signaling required PKA. We tested the ability to rescue the *AKAP200* LOF phenotype of AKAP200-L, which binds PKA, and AKAP200-S, which does not as it lacks the PKA interaction domain (Fig 3A). Strikingly, ubiquitous expression of each isoform (under *tubulin-Gal4* control; Fig 3D and 3E) rescued the *AKAP200* photoreceptor number defects (Fig 3C). This suggests that eye phenotypes are not related to PKA (quantified in Fig 3B; this also confirmed that the mutants are clean *AKAP200* alleles; see also S3 Fig). Similarly, both isoforms were capable of rescuing the *AKAP200* bristle defects (S3K, S3M and S3O Fig). To lend further support to the hypothesis that AKAP200’s supernumerary bristle phenotype can be attributed to Notch signaling, we added an extra copy of Notch using N-GFP,Cherry flies [68] in *an AKAP200* mutant background. Here as well we observed a rescue of the *AKAP200* bristle defects (S3N and S3O Fig).

We also assessed the potential direct involvement of PKA in promoting Notch signaling, and asked whether *sev-N*^*ΔECD*^ (Fig 3G) is sensitive to PKA levels. Removing one genomic copy of *PKA* did not modify the *sev-N*^*ΔECD*^ phenotype (Fig 3H). Moreover, simultaneous removal of one copy each of *AKAP200* and *PKA* (Fig 3J) had the same effect on *sev-N*^*ΔECD*^ as removing only *AKAP200* (Fig 3I; quantified in Fig 3F). To lend further support to this hypothesis, we tested for potential effects of AKAP200-L and AKAP200-S on the *sev-N*^*ΔECD*^*/+* eye phenotypes (S3C Fig); *sev-Gal4* driven overexpression of either AKAP200-L (S3D Fig) and AKAP200-S (S3E Fig) both enhanced *sev-N*^*ΔECD*^ to comparable extents (quantified in S3F Fig); as overexpressing AKAP200-L or S alone caused no defects in the eye (although each of them did in the wing, S3A and S3B Fig), this indicated that in the eye the enhancement is not an additive effect of unrelated phenotypes. Finally, overexpression of either Notch itself (S3H Fig), AKAP200-L (S3I Fig) or AKAP200-S (S3J Fig) in the entire wing blade (*nubbin-Gal4* control) produced similar phenotypes of expanded wing veins.

Taken together, these data are consistent with the notion that AKAP200’s positive role on regulation of Notch signaling is PKA independent.

### AKAP200 stabilizes Notch protein

**Fig 4:**
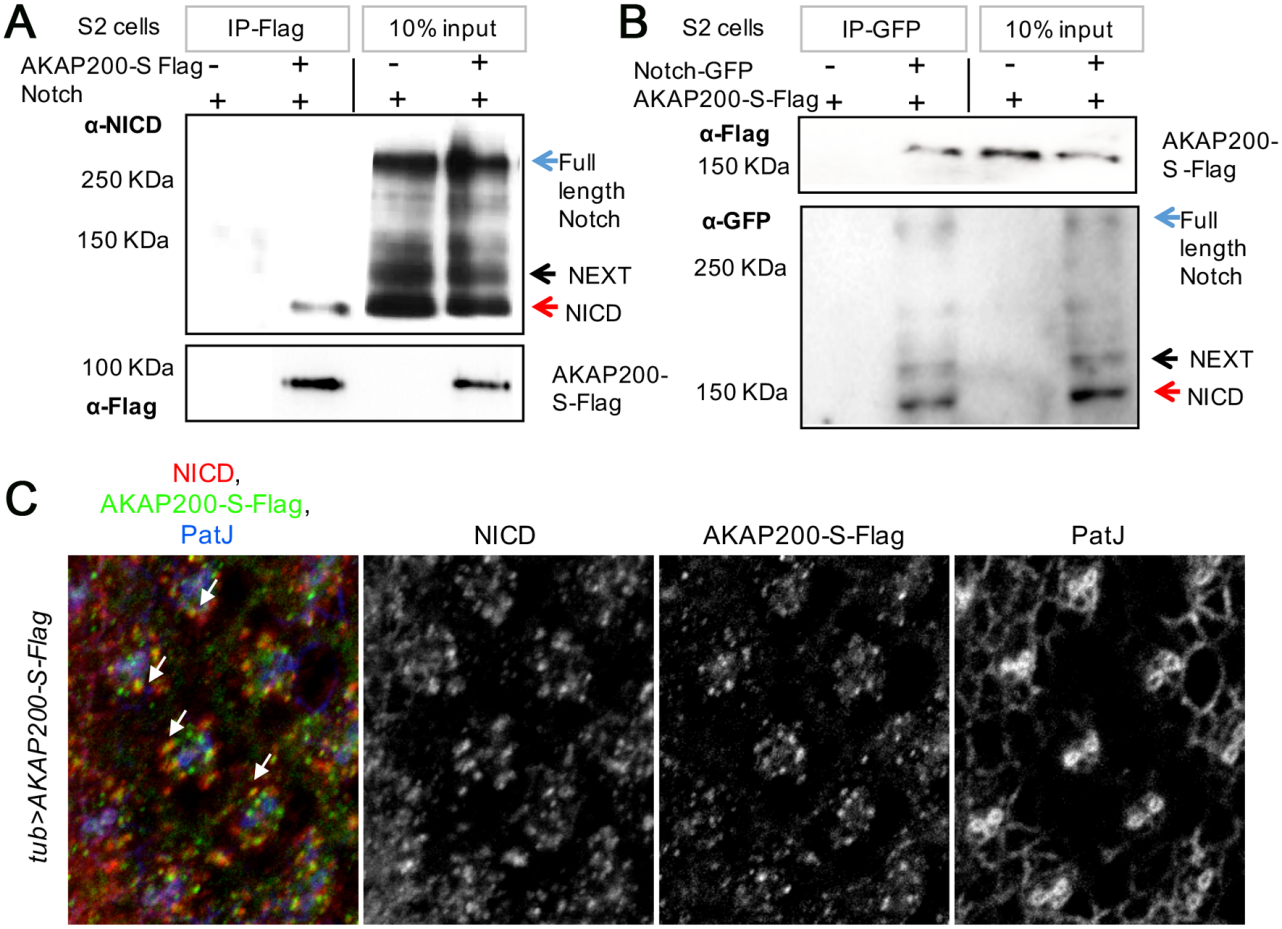
Association of AKAP200 and Notch. (A) Notch is co-immunoprecipitated by AKAP200-S: immunoblot from S2 cell whole cell lysates expressing Notch either in combination with Flag-control or AKAP200-S-Flag. Cell lysates were immunoprecipitated with anti-Flag antibody (IP-Flag) and blots were probed with anti-NICD antibody, revealing specific co-IP of Notch with AKAP200-S-Flag with no binding to Flag (right panel-10% input, bottom panel- blots probed with anti-Flag antibody). The specific interaction of AKAP200 and the NICD could be because of the experimental conditions; full length Notch is a large membrane bound protein (270 KDa) it may not be as easily accessible to AKAP200 as the NICD. Given the large size of full length Notch, one cannot exclude the possibility that the physical conformation of the interaction prevents co-immunoprecipitation; for example, AKAP200 maybe buried inside full length Notch. (B) AKAP200-S is coimmunoprecipitated by Notch: immunoblot from S2 cell whole cell lysates expressing AKAP200-S-Flag in combination with GFP-control or Notch-GFP. Cell lysates were immunoprecipitated with anti-GFP antibody (IP-GFP) and blots were probed with anti-Flag antibody, revealing specific co-IP of AKAP200-S-Flag with Notch with no binding to GFP (right panel-10% input, bottom panel- blots probed with anti-GFP antibody). (C) Confocal eye sections from third larval instar eye discs of *tub>AKAP200-S-Flag* depicting localization AKAP200-S, Notch, and PatJ (marking cellular outlines at junctional level and highlighting developing PR clusters, with strongest staining observed in R2/R5). Note co-localization between anti-NICD punctae and AKAP200-S-Flag (example marked by white arrow), Pearson co-efficient R = 0.67.

Next, we investigated whether AKAP200 and Notch can be present in the same protein complex. Since both AKAP200 isoforms are equivalent with respect to modulating Notch function, we performed our analyses with AKAP200-S only. A Notch encoding plasmid was transfected into S2 cells with either AKAP200-S-Flag or Flag alone. Immunoprecipitation with the Flag antibody led to the co-immunoprecipitation (co-IP) of the NICD fragment in the AKAP200-S-Flag sample but not the control (Fig 4A, schematic of Notch protein in S4B Fig). Long exposure revealed also co-immunoprecipitation of the NEXT with AKAP200-S, but we did not detect immunoprecipitation of full length Notch (S4A Fig). In an inverse experiment, an AKAP200-S-Flag encoding plasmid was transfected into S2 cells with either Notch-GFP or GFP alone. Immunoprecipitation with the GFP antibody led to the co-immunoprecipitation (co-IP) of AKAP200-S-Flag in the Notch-GFP sample but not the control (Fig 4B).

To confirm the physiological relevance of these results, we examined the localization of AKAP200-S-Flag and endogenous Notch in third instar eye and wing imaginal discs. We observed co-localization of AKAP200 and Notch (Fig 4C, co-stained with PatJ, R = 0.67, S4C Fig, co-stained with E-Cad). Similarly, we observed colocalization of the two proteins in the portion of the wing imaginal discs from which the thorax arises (S4D Fig, R = 0.4).

**Fig 5:**
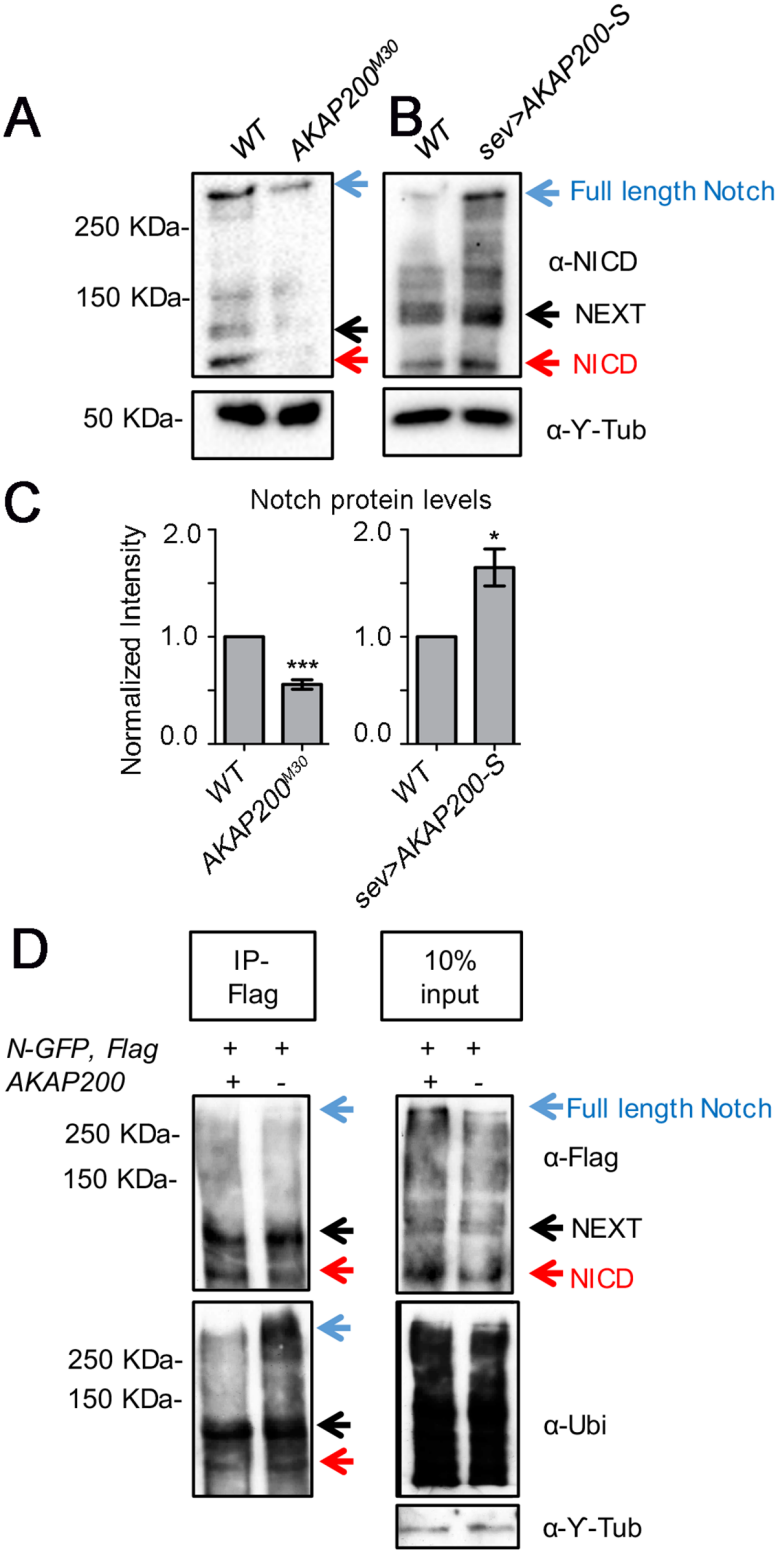
AKAP200 affects Notch levels and ubiquitination. (A-B) Western blots of third instar larval eye disc extracts using NICD and gamma tubulin antibodies [blue arrow: full length Notch; black arrow: membrane bound NEXT; red arrow: NICD, [100]]. (A) In *AKAP200*^*M30*^ samples, note a decrease in Notch protein levels compared to *WT* (loading control:©-Tubulin [Y-Tub], bottom here and all other panels). (B) Over-expression of AKAP200-S (by *sev-Gal4*) leads to an increase in Notch levels, compared to *WT*. (C) Quantification of Notch protein levels in (A-B) (n = 3, ***p<0.0001, *p = 0.01 by student’s t test; error bar = standard deviation). (D) Increased ubiquitination of Notch is observed in *AKAP200*^*M30*^ mutant. Eye disc lysates from *Notch-Flag/+* or *Notch-Flag/+*, *AKAP200*^*M30*^*/+* were immunoprecipitated with anti-Flag and probed with anti-Flag and anti-Ubiquitin. Leupeptin and protease inhibitors were added to the lysis buffer. Blotting with anti-Ubiquitin revealed an increase of ubiquitinated full length Notch, NEXT and NICD in the *AKAP200* mutant.

To dissect the mechanism(s) underlying the genetic interactions between *AKAP200* and *Notch* and as AKAP200 promotes Notch signaling, we tested whether AKAP200 could regulate Notch protein cleavage or levels. We did not observe any reproducible changes to the Notch cleavage patterns (examples in Fig 5A and S5A Fig). However, strikingly, total endogenous Notch levels were markedly reduced in homozygous *AKAP200* mutant backgrounds as compared to *WT* (Fig 5A and 5C; total Notch levels are the sum of full length Notch, transmembrane Notch/NEXT, and NICD, S5A Fig). Conversely, overexpression of AKAP200-S caused an increase in Notch levels (Fig 5B and 5C). Furthermore, RT-PCR amplification of *Notch* showed no significant gene expression differences in *AKAP200*^*M30*^ eye disc lysate relative to *WT* (S5B Fig). Several studies have demonstrated both lysosomal and proteosomal degradation of Notch [20–24]. Thus, we postulated that AKAP200 might regulate Notch turnover. To test this hypothesis, we asked if there is differential ubiquitination of Notch in *AKAP200* mutants relative to *WT*. We performed immunoprecipitations of Notch-Flag from eye disc lysates of *WT* and *AKAP200*^*M30*^*/+* larvae. Upon immunoprecipitation with Flag (and thus Notch) and probing for ubiquitin, there was an increase in ubiquitinated Notch-fl, NEXT and NICD fragments in *AKAP200*^*M30*^*/+* backgrounds (Fig 5D). These observations suggest that AKAP200 promotes Notch signaling by stabilizing Notch protein levels and are consistent with the genetic interaction data and corroborated by the observation that 3 genomic copies of *Notch* rescue *AKAP200* LOF defects.

### AKAP200 negatively regulates Cbl-mediated lysosomal degradation of Notch

In C2C12 myoblasts, Notch has been shown previously to be targeted for lysosomal degradation as a consequence of mono-ubiquitination by the E3 ubiquitin ligase, Cbl [21]. We thus hypothesized that the reduction of Notch levels and its increased ubiquitination in *AKAP200* mutants could involve Cbl.

**Fig 6:**
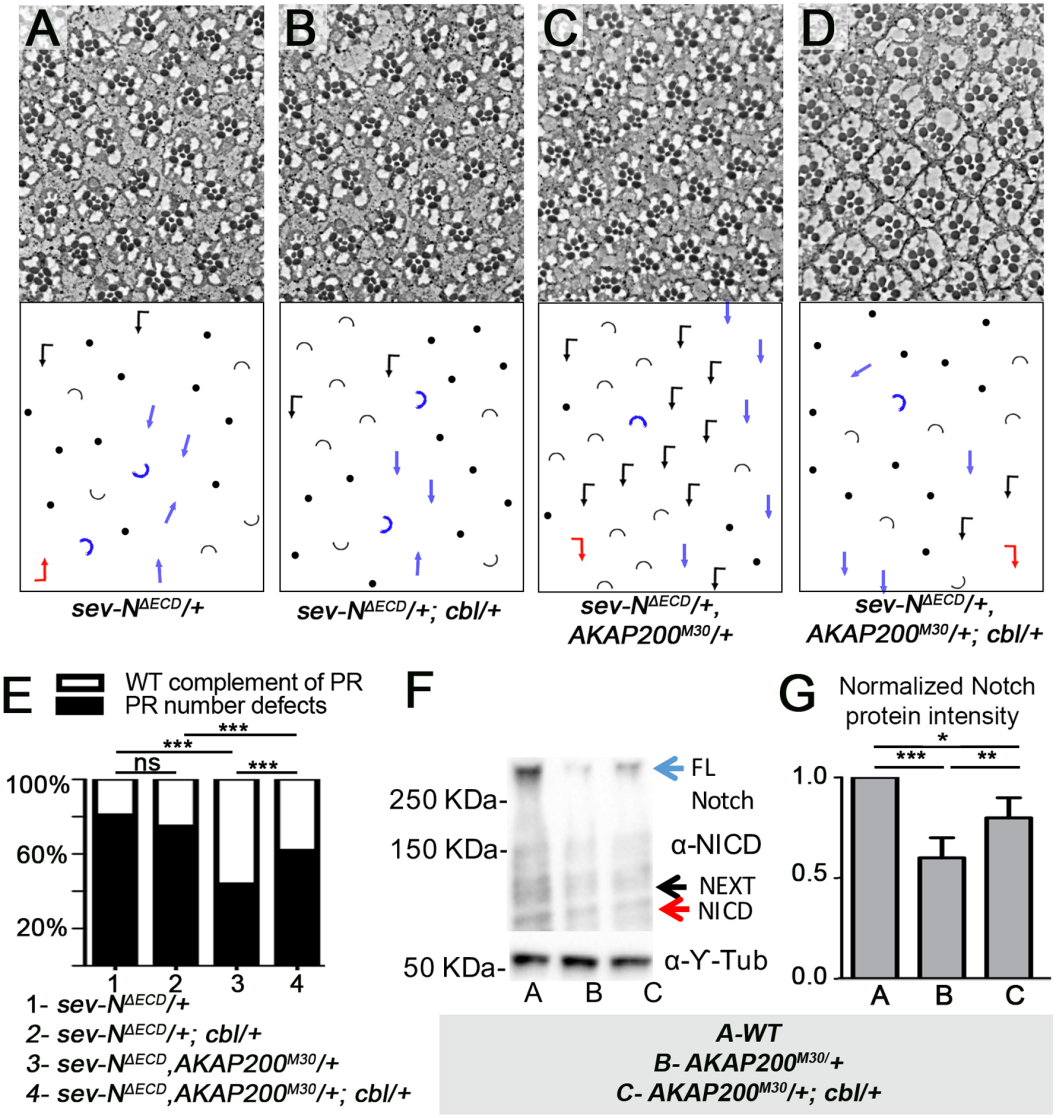
The effect of AKAP200 on Notch signaling depends on Cbl. **(A-D) Tangential adult eye sections of indicated genotypes, with schematics in lower panels (see** Fig 2A **for key).** (A) PR number defects induced by *sev-N*^*ΔECD*^ are not modified by the heterozygous *cbl* mutant alone (B), but are suppressed by heterozygous *AKAP200* mutant (C). (D) Simultaneous reduction of one genomic copy of both *cbl* and *AKAP200* reduces the effect of *AKAP200* suppression of Notch activation. (E) Quantification of genetic interactions of genotypes in (A-D): ***p<0.0001 from chi square tests (n = 320–686 from 3–4 independent eyes). (F-G) Western blot (F) of third instar larval eye disc lysate showing Notch protein expression (blue arrow: full length Notch, black arrow: membrane bound NEXT, red arrow: NICD). Relative to *WT* (left lane), a reduction in total endogenous Notch protein is observed in *AKAP200*^*M30*^*/+* (middle lane), which is partially suppressed in lysates from *AKAP200*^*M30*^*/+; cbl/+* (©-Tub [Y-Tub], bottom, serves as loading control). (G) Quantification of Western blot lanes from (F); n = 3, **p = 0.003, p = 0.01 from student’s t test (error bars represent standard deviations).

To explore a role for *cbl* in the interplay between *AKAP200* and *Notch*, we first tested whether they interacted genetically. Removing one genomic copy of *cbl* (Fig 6B) did not modify the *sev-N*^*ΔECD*^*/+* phenotype (Fig 6A). However, the *AKAP200*^*M30*^*/+* suppression of *sev-N*^*ΔECD*^*/+* (Fig 6C) was dependent on Cbl levels, with the suppression effect being markedly reduced upon simultaneous removal of one copy of both *AKAP200* and *cbl* (Fig 6D, quantified in Fig 6E). This suggests that the positive effect of AKAP200 on Notch levels and signaling activity is through antagonizing the negative function of Cbl.

To confirm this, we examined the interaction between *AKAP200*, *cbl* and *Notch* in SOP specification, activating the pathway with the *H*^*1*^ allele and using bristle loss as the GOF assay (quantified in S6A Fig, depicted in S6B Fig). Consistent with the eye data, *AKAP200*^*M30*^*/+* suppressed the *H*/+ phenotype (S6D Fig, quantified in S6A Fig). Simultaneously removing one copy of both *AKAP200* and *cbl* (S6E Fig) dampened the effect of *AKAP200’s* loss on the *H*/+ phenotype (S6D Fig, quantified in S6A Fig; note that *cbl*/+ alone as a control has no effect on *H*^*1*^*/+*, S6C Fig). As previously observed (S2F and S2I Fig), *AKAP200*^*M30*^/+ enhanced the *N*^*55e11*^/+ scutellar phenotype (S6I Fig), and consistently with the above interactions, simultaneous removal of a genomic copy of both *AKAP200* and *cbl* limited the effect of *AKAP200*^*M30*^/+ on *N*^*55e11*^/+ (S6J Fig; note that *N*^*55e11*^/+; *cbl*/+ alone as a control has no effect S6G Fig). Taken together, these data are consistent with the notion that AKAP200 antagonizes Cbl to promote Notch stability and hence promote signaling.

The Cbl docking consensus site has been mapped to the vicinity of Notch’s PEST domain (S4B Fig). We thus expressed a Notch isoform truncated at amino acid 2155 [69] and thereby deleting the PEST domain (under *sev-Gal4* control) either in *WT* or in the *AKAP200*^*M30*^ background. Unlike with full-length Notch, we detected no significant difference in the phenotypic effect between the two genotypes (S6K and S6L Fig, quantified in S6M Fig). This suggests that the role of AKAP200 in promoting Notch function depends on the presence of the PEST domain, consistent with the hypothesis that AKAP200 protects Notch from Cbl-mediated ubiquitination.

We next assessed the effects of AKAP200 on Cbl-mediated Notch level reduction. We compared Notch protein levels from *WT* eye discs to *AKAP200*^*M30*^*/+* and *AKAP200*^*M30*^*/+; cbl*/+ discs. While reducing the copy number of *AKAP200* resulted in a decrease of Notch levels, concurrent reduction in copy number of both *AKAP200* and *cbl* largely abolished this effect (Fig 6F, quantified in Fig 6G). In line with this observation, *Cbl/+* suppresses the *AKAP200*^*M30*^ PR number defect phenotype (S6N Fig).

To confirm specificity of the AKAP200 effect on Cbl, we tested other E3-ubiquitin ligases for an interaction with *AKAP200*. The *Drosophila* homolog of Sel10/Fbw7 E3-ligase, *archipelago* (*ago*), is an E3 ligase that also has been shown to ubiquitinate NICD and target it for proteosomal degradation [19]. Since *ago* is also a transcriptional target of Notch [70], the effect of removing one copy of *ago* likely affects feedback loops, and thus was not included in our analyses. Instead, we tested if the *AKAP200* effect on Notch can be altered if a copy of *ago* is also removed together with *AKAP200*. Simultaneous removal of one copy of both, *ago* and *AKAP200*, affects the *sev-N*^*ΔECD*^*/+* phenotype to the same extent as removing only a copy of *AKAP200*, implying that *AKAP200* and *ago* act via unrelated mechanisms (S6M Fig). This suggests that *AKAP200*’s effect is specific to Cbl’s ubiquitination of Notch.

**Fig 7:**
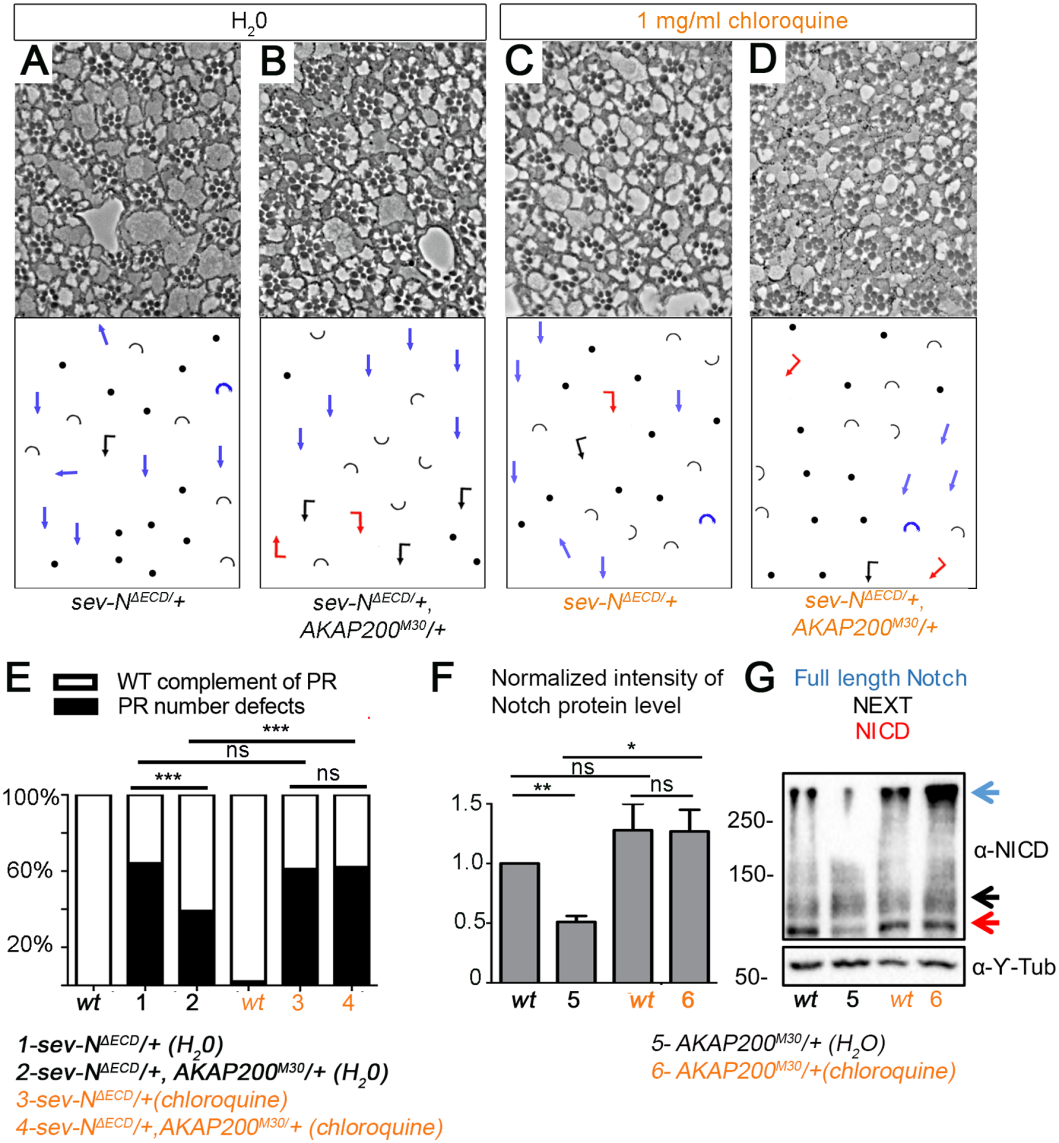
AKAP200 effect on Notch depends on lysosomal degradation. (A-D) Tangential adult eye sections of indicated genotypes and conditions, and (E) quantification of indicated genotypes/conditions; suppression of PR number defects of *sev-N*^*ΔECD*^*/+* by *AKAP200* is lost in the presence of 1mg/ml of lysosomal inhibitor, chloroquine (***p<0.0001 from chi square tests, n = 378–644 from 3–4 independent eyes). (F) Quantification (**p = 0.005, *p = 0.02, n = 4) and (G) western blot of third instar larval eye discs showing Notch protein levels in indicated genotypes. Relative to *WT*, heterozygous *AKAP200* mutant decreases Notch levels (under control condition, H_2_0 treatment), this effect is lost in the presence of 1 mg/ml chloroquine (Y-Tub, bottom, serves as loading control).

Since AKAP200 appeared to protect Notch against the effects of Cbl, which targets Notch to the lysosome, we wanted to investigate the requirement of the lysosome in AKAP200’s action on Notch. We thus analyzed the effect of *AKAP200* on *sev-N*^*ΔECD*^ in the presence of the lysosomal inhibitor chloroquine, which is a lysosomotropic agent acting by increasing lysosomal pH thus inhibiting lysosomal hydrolases as well as fusion of endosomes and lysosomes and thereby impairing degradation [71–74]. The effect of *AKAP200*^*M30*^/+ on *sev-N*^*ΔECD*^*/+* under control conditions (Fig 7A and 7B, quantified in Fig 7E) was lost when larvae were raised on 1mg/ml chloroquine (Fig 7C and 7D, quantified in Fig 7E). At this dosage, chloroquine by itself had negligible effects on normal development and cell viability, and PR number (quantified in Fig 7E, S7F Fig). Also, the average lifespan of both *sev-N*^*ΔECD*^*/+* and *sev-N*^*ΔECD*^*/+*, *AKAP200*^*M30*^*/+* were comparable at increasing exposures to chloroquine; survival of both genotypes dropped only at chloroquine concentrations significantly greater than 1mg/ml (S7F Fig; confirming a specific effect at 1mg/ml chloroquine and not a general interference). Immunofluorescence analyses in the larval eye disc showed minimal, if any, colocalization of AKAP200 and the lysosome (S7G Fig). This is consistent with the notion that AKAP200 acts on Notch to prevent its targeting to the lysosome by antagonizing Cbl-mediated ubiquitination of Notch which occurs before Notch is targeted to the lysosome.

Importantly, reduction of Notch protein levels in the heterozygous *AKAP200* mutant background, relative to *WT*, was lost upon chloroquine treatment (Fig 7G, quantified in Fig 7F). This highlights AKAP200’s dependence on the lysosome to promote Notch signaling and is consistent with its antagonistic effect on Cbl-mediated ubiquitination of Notch.

To confirm these results, we conducted analogous experiments in the context of SOP specification. *AKAP200/+* suppression of the *H/+* bristle phenotype observed under control conditions (quantified in S7A Fig, depicted in S7B and S7C Fig), was largely lost upon chloroquine treatment (S7D and S7E Fig, quantified in S7A Fig).

Taken together, our data indicate that the mechanism by which AKAP200 promotes Notch signaling, is by protecting Notch protein from the action of Cbl, which targets Notch for lysosomal degradation.

## Discussion

In this study, we have identified AKAP200 as a positive regulator of the Notch signaling pathway, and carried out a detailed analysis of its LOF phenotypes in eyes and wings using null alleles. We demonstrate that AKAP200 promotes Notch signaling, and that the two proteins co-localize and coexist in a complex. AKAP200 exerts its effects on the Notch protein by protecting it from Cbl/lysosome-mediated degradation in specific contexts, in particular during photoreceptor neuron and sensory bristle specification.

### AKAP200, Notch, and signaling pathway contexts

AKAP200 was identified in a PCP-signaling mediated screen performed in the *Drosophila* eye [39]. However, *AKAP200* LOF phenotypes resemble *Notch* LOF. As PCP is instructive to Notch signaling in the R3/R4 specification context in the *Drosophila* eye, the identification of novel Notch pathway regulators was expected and in line with previous experiments; for example, the Notch ligand *Dl* was identified in the screen as well [39]. Furthermore, *AKAP200*’s strong and specific interaction with *sev-N*^*ΔECD*^, which is a membrane tethered, ligand-independent activated Notch, indicates that it acts on Notch itself.

Due to AKAP200’s ‘canonical’ role in PKA regulation, we tested how AKAP200 acts in Notch signaling. Analyses of the two isoforms of *AKAP200*, which differ in their ability to bind PKA, revealed that AKAP200’s Notch associated function is PKA independent, which was corroborated by functional rescue assays with both isoforms rescuing the *AKAP200* eye phenotypes indistinguishably. The *AKAP200* LOF mutants display other defects, which in some tissues are PKA associated phenotypes: for example *AKAP200* mutant wings have a penetrant wing blistering phenotype, which has been observed upon disruption of the PKA pathway [59, 60] and, similarly, *AKAP200* mutant ovaries have developmental defects, which have been linked to PKA signaling [75].

Our work identifies AKAP200 as a regulator of Notch protein levels (also below). However, it does not affect Notch in all tissues, and even in tissues where it is required, it is specific to a subset of Notch signaling contexts. In the eye for example, it affects Notch signaling during photoreceptor specification but not during lateral inhibition in the furrow. Strikingly, there are no effects of *AKAP200* on Notch signaling mediated wing margin development, which is even a haplo-insufficient Notch phenotype [76, 77]. Likely, the Notch signaling feedback loops at the wing margin, which also includes Wingless (Wg) expression [78–80], are not sensitive to AKAP200 mediated input. However, overexpression of AKAP200 in the wing led to expansion of wing veins, a phenotype linked to Notch GOF [81, 82], and thus consistent with the Notch GOF effects in photoreceptor specification in the eye. Taken together, our data suggest that AKAP200 affects Notch levels in a tissue and context specific manner, rather than being a general Notch protein level regulator.

### AKAP200 promotes a subset of Notch signaling events

How do the AKAP200 Notch signaling requirements relate to each other? In the eye, *AKAP200* LOF defects correlate with photoreceptor specification, particularly with R7 and R4 induction, and associated steps. Specification of R7 and R4 both require Notch signaling activation from neighboring R-cells, R1/6 and R8 induce R7 and R3 activates the pathway in R4, and interestingly in both contexts Egfr/RTK signaling is also required for the specific R-cell fate [30, 33, 36–38, 83]. We did not detect an interaction between AKAP200 and the GOF *Egfr*^*Ellipse*^ alleles, however, suggesting that AKAP200 does not act via Egfr.

Can this correlation be related to other AKAP200 requirement contexts? In the wing, although there is no *AKAP200* effect on the margin, both overexpression and LOFs of AKAP200 affect wing vein development. Establishment of wing veins is a multi-step, multi-pathway process, involving coordination of Notch signaling and other pathways, which also include Egfr/RTK signaling [84–88]. Notch signaling causes restriction of cell fate and width of the vein [4, 66, 89–92]. Consistent with the defects in the eye, LOF or GOF of AKAP200 correlates with vein development or vein widening, respectively.

In the thorax, Notch is required at different stages of SOP specification and *AKAP200* LOF phenotypes resemble several of these. Can this be linked to Egfr signaling as well? Previous reports have shown an involvement of Egfr/RTK signaling in promoting bristle development, where *Egfr* hypomorphs developed fewer bristles [93, 94]. The *Egfr* requirement has been attributed to SOPs requiring Egfr-signaling to maintain wild-type levels of *ac-sc* expression [95].

In summary, it appears that AKAP200 affects Notch activity/levels in specific contexts and that these involve Egfr signaling in some capacity.

### AKAP200 prevents Cbl-mediated ubiquitination and degradation of Notch

The role of AKAP200 appears to be in stabilization of the Notch protein: there is a decrease of endogenous Notch in the *AKAP200* LOF vs. an increase of Notch in AKAP200 GOF backgrounds. We also observed increased ubiquitination of Notch in *AKAP200* null mutant backgrounds. Ubiquitination and subsequent degradation of cellular proteins serves as a key mechanism to regulate their activity and disruption in this process often lead to overactivation of signaling. Both AKAP200 and Notch have previously been associated with the E3 ubiquitin ligase, Cbl [21, 96], and strikingly Cbl has also been linked to the regulation of Egfr [97, 98]. Thus, we explored a role for Cbl in AKAP200’s regulation of Notch. Strikingly, suppression of Notch hyperactivation by AKAP200 depends on the presence of wild-type levels of Cbl. Our studies thus suggest that AKAP200’s function is to antagonize Cbl effects on Notch ubiquitination and protein levels. Of note, it is also possible that AKAP200 modulates Notch or other components of the signaling pathway via other mechanisms.

One hypothesis that leads from our work is that AKAP200 could maintain the balance between Cbl’s effects on Notch and Egfr. Since AKAP200 is a scaffolding protein, it may affect both pathways, and only processes that require balanced effects of Notch and Egfr signaling may be impacted. AKAP200 was previously identified as a positive regulator of Ras signaling [99]. However, we did not pursue AKAP200’s role in this context as we did not detect interactions with the *Egfr*^*Elp*^ allele.

In conclusion, we postulate a novel mechanism of regulation of Notch signaling by AKAP200 antagonizing Cbl-mediated lysosomal degradation of Notch. This study advances our understanding of the tight regulation of Notch protein levels, which is fundamental to numerous key developmental processes and diseases.

## Materials and methods

### *Drosophila* strains and genetics

Flies were raised on standard medium and maintained at 25°C unless otherwise indicated. All *WT* experiments were performed in *w*^*1118*^ backgrounds.

The following stocks lines were used and their sources are indicated:

*Df(2L)Ed623* (stock #8930), *Df(2L)N22-14*(stock #2892), w1118, *N*^*55e11*^ (stock #28813), *PKA-C1*^*H2*^ (stock #4101), *UAS-Notch* (stock #26820), *cbl*^*F165*^ (stock #9676), *Notch-GFP*,*Flag* (stock #BL38665), *ago*^*EY01092*^ (stock #20064)—Bloomington stock collection
*UAS-AKAP200-IR* (stocks- 1- #5647, 2- #102374)–VDRC
*Elp–*gift from Dr. Ross Cagan, *sev-N*^*ΔECD*^*–*gift from Dr. Mark Fortini, *mδlacZ* [37], *UAS-Notch*^*ΔTAD*,*PEST*^- gift from Dr. Edward Giniger, *H*^*1*^ [101], *Su(H)*^*Δ47*^ [102], *UAS-Dgo;sev-Gal4* [39], *Actin-Gal4;UAS-GFP-huLamp–*gift From Dr. Andreas Jenny, *N-GFP*,*Cherry*–gift from Dr. Francois Schweisguth [68].

The Gal4/UAS system [103] was employed for gene expression studies and the following Gal4 drivers were used: *sep-Gal4* [104], *sev-Gal4* [63], *tub-Gal4, nub-Gal4* (Bloomington stock center).

*sev-gal4* (*sev*-enhancer with *heat-shock* promoter) initially drives expression in the R3/R4 precursor and later in R1/6 and R7 (note, there is basal expression in other tissues due to the presence of the heat shock promoter from *hsp70)*. *sep-gal4 which has the sev*-enhancer with *sev* promoter, results in weaker expression levels than *sev-gal4*.

AKAP200^M30^ clones were produced by mitotic recombination via the FLP/FRT system *[47]* with *eyFLP* in an AKAP200^M30^ FRT40A/ w armlacz FRT40A background and *ubxFLP in AKAP200M30FRT40A/ y FRT40A* background.

AKAP200^M30^ and AKAP200^M24^ were generated using a FLP-recombinase-mediated excision of two *piggybac/FRT* insertions grk^f07069^ and AKAP200^d03938^ and characterized by PCR as previously described [46]. To generate UAS-AKAP200-Flag transgenic flies, the Flag tag was added to the C-term of AKAP200 sequence by PCR amplification using DGRC LD42903 and RE01501 cDNA clones for AKAP200-L and AKAP200-S respectively. The PCR amplified products were cloned into pUASt-attB vector using EcoRI and XhoI sites.

The following primers were used: 5’-CCGGAATTCATGGGTAAAGCTCAGAGCAA-3’and 5-CCGCTCGAGCTTGTCGTCGTCGTCCTTGTA-3’

Transgenic injections were performed by BestGene Inc. where the constructs were targeted to predetermined genomic sites on chromosome 3R using the phiC31 integrase (strain 9744).

For drug treatments, crosses were setup on instant food (Carolina Biological Supply Company) to which chloroquine diphosphate salt (Sigma) or water was added at the indicated concentrations.

### RT-PCR

To compare relative amount of *Notch* mRNA, RNA was extracted from eye disc lysates from *WT* or *AKAP200*^*M30*^ flies using RNeasy Mini Kit as per manufacturer’s protocol (Qiagen). 1 ng of RNA was reverse transcribed (50°- 30’ and 94°- 2’) and real-time PCR was performed using SYBR Green I Master (Roche) on LightCycler 480 (Roche). Quantification was performed using the 2-ΔΔCT method and *Gapdh* transcript as a reference. Measurements were performed in duplicate. The following primers were used:

rp49–5’-GACAGTATCTGATGCC-3’ and 5’- TTCCGACCACGTTACAAGAAC-3’
Notch- 5’-GAGTGGAGCCGGCAATGGAAAT-3’ and 5’-TTCAAAACCTACAGAACTACGA-3’

The amplified products are expected to be ~300 bp for rp49 and ~1600 bp for Notch. As control for DNA contamination in eye disc lysates, a reaction was run using Notch primers excluding reverse transcriptase (rxn mixture).

### Immunofluorescence and histology

Third larval instar eye discs were dissected in ice cold PBS and fixed in PBS-4% paraformaldehyde for 20 minutes at room temperature. After three washes in PBT (PBS + 0.1% Triton-X), discs were incubated in primary antibody overnight at 4°C. After three PBT washes, secondary antibody incubation for 2 hours at room temperature and three more PBT washes, the discs were mounted in Vectashield (Vector Laboratories). For immunofluorescence, the following antibodies were used- mouse anti-Prospero (#MR1A, 1:10, Developmental Studies Hybridoma Bank-DSHB), rat anti-Elav (#9F8A9, 1:20, DSHB), mouse anti-Notch^ICD^ (#C17.9C6, 1:10, DSHB), rabbit anti-Flag (#637301, 1:100, Biolegend), mouse anti-Flag (#F1804, 1:1000, Sigma), rat anti-DE Cadherin (#5D3, 1:20, DSHB), rabbit anti-Patj (1:500), rabbit anti-GFP (#1828014, 1:1000, invitrogen), rabbit anti-β-gal (1:1000, Molecular probes). Fluorescent secondary antibodies came from Jackson Laboratories. Eye disc and thorax images were acquired at room temperature using a Zeiss LSM 880 or Leica SP5 DMI confocal microscopes Subsequent image processing was performed on ImageJ (National Institute of Health). For colocalization analyses, the JaCoP plugin was used in ImageJ to calculate the Pearson’s coefficient (R).

Eye sections were prepared as previously described [105]. All eyes were sectioned near the equatorial region. For analysis of adult thoraces, whole flies were incubated in 70% ethanol and mounted on gelatin plates. Imaging was done using a stereomicroscope and acquired using Zeiss Axioplan color type 412–312 (Carl Zeiss) camera and Zen Blue software. For analysis of adult wings, wings were removed and incubated in PBT, mounted on a slide in 80% glycerol and imaged using a Zeiss Axioplan microscope (Carl Zeiss).

### Western blotting and co-immunoprecipitation

For analysis of Notch protein levels, 10–15 pairs of larval eye discs were lysed in ice cold lysis buffer (50mM Tris HCl pH 7.5, 150mM NaCl, 1mM EDTA and 1% Triton-X). Supernatant from these extracts were resolved and subjected to standard western blotting procedures using mouse anti-Notch^ICD^ (#C17.9C6, 1:500, DSHB) and mouse anti-Y-Tubulin (Sigma Aldrich, 1:1000) antibodies.

For co-immunoprecipitation assays, S2 cells were transfected with pmt-Notch^full length^ (#1022 from DGRC) with pAC-Flag control or pac-AKAP200-S-Flag, or pac-AKAP-200-S-Flag with pUAST-GFP or pUAST-Notch-GFP (gift from Dr. Shigeo Hayashi), both with Actin-Gal4. Transfections were performed using Effectence (QIAGEN, Hilden, Germany) in accordance with manufacturer’s instructions. For pmt-Notch, Notch was induced using 600 μM CuSO_4_ 24 hours after transfection for 24 hours [100].

For Flag IPs and ubiquitin assays, cells were harvested, washed and lysed in ice-cold lysis buffer (50mM Tris HCl pH 7.5, 150mM NaCl, 1mM EDTA and 1% Triton-X). For ubiquitin assays, the lysis buffer was supplemented with 100 μg/mL leupeptin (Roche 1017101) and protease inhibitor cocktail tablets (Roche, 14268500).

For Flag IPs, lysed samples were incubated overnight at 4°C using anti-Flag M2 agarose beads (Sigma, A2220). Beads were washed three times and protein was eluted by boiling in Laemmli buffer.

For GFP IPs, cells were harvested, washed and lysed in ice-cold lysis buffer (0mM TrisHCl pH7.5, 150mM NaCl, 0.5mM EDTA, 1% TritonX). Following this, we used the GFP-Trap A from Chromotek as per manufacturers protocol (wash buffer: 10mM TrisHCl pH7.5, 150mM NaCl, 0.5mM EDTA).

Western blots were carried out with the immunoprecipitated samples using mouse anti-Notch^ICD^ (#C17.9C6, 1:500, DSHB), mouse anti-Flag (#F1804, 1:1000, Sigma), mouse anti-Ubiquitin (#MA1-10035, 1:5000, Thermofisher) and rabbit anti-GFP (#1828014, 1:1000, Invitrogen). HRP coupled secondary antibodies were obtained from Jackson laboratories.

## Supporting information

S1 Fig *AKAP200* shows N-signaling like phenotypes in the Drosophila eye and thorax. (A-B) AKAP200 was identified in a dominant modifier screen. (A) Table shown summarizes modifications of the core PCP factor *dgo* by *AKAP200* deficiencies and IR (*dgo* was overexpressed by *sev-Gal4* which has the *sev* enhancer and *hs* promoter leading to strong expression in R3, 4, 1, 6, 7 and cone cells, and basal expression in other tissues including the wing). (B) Quantification of genetic interactions in adult eyes: *AKAP200*^*IR*^ enhances rotation defects from *dgo* overexpression (***p<0.001 from chi square test; n = 536–754, 3–4 independent eyes). (C-D) *AKAP200* mutant generation and characterization: schematic of locus (C), grey bars represent coding exons of the gene. Transposable elements used to generate null mutants, *P(AKAP200*^*d03938*^*)* and *Pbac(grk*^*f07069*^*)* hereafter called XP5 and WH5 respectively, are indicated in orange and green, and flank the gene. Precise excision results in fusion of the elements and elimination of *AKAP200* coding sequences. For PCR characterization, a primer pair was used that sits on elements XP5 and WH5 (depicted by black arrows), or directly outside XP5 and WH5 (depicted by blue arrows). Due to the genomic distance between the primers, in both cases, PCR amplification is only possible if the excision event happened. The expected band size for amplified band when primer combination sitting on XP5 and WH5 is used is ~1.5 Kb and for the primers outside these elements is ~1.7 Kb. (D) PCR characterization of *AKAP200* null mutants from genomic DNA extracted from adult escaper flies of indicated genotypes. For lanes labeled in black, primers used sit within the transposable elements XP5 and WH5; for lanes labeled in blue, primers used sit directly outside XP5 and WH5. XP5 and WH5 are absent in WT DNA resulting in no PCR amplification, and the primers outside these elements are too far to result in PCR amplification in WT DNA. Genomic DNA from two null mutants *AKAP200*^*M30*^ and *AKAP200*^*M24*^ give a band at the expected sizes of ~1.5–1.7 Kb upon PCR amplification. (E-F) Pupal thorax clones stained for Elav (white) and Cut (red); mutant tissue marked by absence of green marker. Note irregular spacing of SOPs and “bald” patches (yellow arrows) as evident by Elav staining in (E-E’). Higher magnifications (F-F”) reveal defects in SOP lineages with clusters containing two Elav positive cells (yellow arrowheads) or reduced Elav staining (blue arrowhead). (G) Quantification of distribution of eye phenotypes upon AKAP200 knockdown by two independent IRs or from transheterozygous *AKAP200* null mutant/*AKAP200* deficiency. White indicates WT, blue indicates loss of one or more outer PRs, green indicates loss of R7 with or without simultaneous loss of outer PRs. Of note, *AKAP200* null mutant/*AKAP200* deficiency have highy reduced viability (<5%). In contrast, overexpression of AKAP200 caused minimal defects. (H) Tangential adult eye sections from example escaper transheterozygous *AKAP200* mutants (genotype as indicated). (I-I’) AKAP200 defects in developing eye discs as revelaed by Elav staining (green) and Pros staining (red). Note defects in Elav positive cells and (I’) loss of Pros+ (R7) cells (examples marked by yellow arrowheads; note that Pros staining in cone cells [blue arrowhead is present and serves as control]). A *wt* looking example is marked by white asterisks. (J) Homozygous *AKAP200*^*M30*^ escaper showing penetrant blistered wing phenotype.
(JPG)

S2 Fig AKAP200 promotes Notch signaling. (A) Quantification of genetic interactions in adult nota by assessing bristle number in indicated genotypes. Activation of the Notch pathway using the *Hairless* (*H*) mutant, the co-repressor that keeps Notch target genes off in the absence of signal, results in decreased number of bristles compared to *WT*. This phenotype is dominantly rescued by removal of one copy of *Su(H)[Su(H)*^*Δ47*^, *null allele]* or *Notch (N*^*55e11*^, null allele). Similar suppression is observed with *AKAP200* mutants (M30 and M24) and deficiency, note *AKAP200*^*M30*^*/+* has no phenotype (***p<0.0001, *p = 0.02 by Mann Whitney test against *H*^*1*^*/+* from 5–20 flies). (B-E) Examples of adult heads as representations of total bristles of indicated genotypes, loss of bristles is indicated by blue asterisk. (B) *WT* head showing normal bristle arrangement. (C) *H*^*1*^*/+* head showing loss of bristles. (D) *H*^*1*^*/+*, *Su(H)/+* and (E) *AKAP200*^*M30*^*/+; H*^*1*^*/+* showing strong and moderate suppression of *H*^*1*^*/+* loss of bristles phenotype respectively. (F) Quantification of genetic interactions in adult scutellum by assessing supernumerary bristles in indicated genotypes. Reduction of Notch signaling in *N*^*55e11*^ null mutant causes increase in number of bristles, which is enhanced by loss of *AKAP200* mutants or deficiency, note *AKAP200*^*M30*^*/+* has no phenotype (***p<0.0001, **p = 0.0016, *p = 0.04 by Mann Whitney test against *N*^*55e11*^*/+* from 9–35 flies). (G-J) Examples of adult scutellar bristles of indicated genotypes, red arrow indicates supernumerary bristles. (G) *WT* scutellum showing normal bristle arrangement. (H) *N*^*55e11*^*/+* showing supernumerary bristles. (I) *N*^*55e11*^*/+; AKAP200*^*M30*^*/+* and (J) *N*^*55e11*^*/+; AKAP200*^*M24*^*/+* show significant enhancement of *N*^*55e11*^*/+* phenotype. (K) Confocal images of third instar eye discs of indicated genotypes stained for neuronal marker Elav (red, labeling all PR cells) and LacZ which stains mδ (Green, initially expressed at low levels in R3 and R4, following Notch activation it is upregulated in R4). One R4/ommatidium is observed in *WT* (top row). Activation of Notch signaling by *sev-N*^*ΔECD*^ increases R4s/ommatidium (middle row), which is suppressed with simultaneous reduction of *AKAP200* (bottom row). (L) Quantification of (K), n = 91–125 ommatidia from 4 independent eyes; ***p<0.0001 by chi square test. (M) Removal of one genomic copy of *AKAP200* has no effect on the PR number defects of EGFR GOF allele *Elp (*chi square tests, n = 547–683 from 4 independent eyes) (N-O) Examples of adult wings of indicated genotypes (N) *Elp/+* displays wing vein overgrowth defect and (O) taking away one copy of *AKAP200* in this background does not this dominant phenotype.
(JPG)

S3 Fig AKAP200 promotes Notch signaling in a PKA independent manner. (A-E) Tangential adult eye sections of indicated genotypes (A) *AKAP200-L* and (B) *AKAP200-S* overexpression under *sev-Gal4* results in <1% defects. *sev-Gal4* driven overexpression produces stronger phenotypes than *tub-Gal4;* both isoforms of AKAP200 produced a negligible effect upon overexpression by *sev-Gal4*. This indicates that *AKAP200* overexpression rescues the mutant with no additive effects of its own phenotype (cf. to Fig 3A). (C-E) The PR number defects induced by *sev-N*^*ΔECD*^ (C) are enhanced by co-expression of *AKAP200*-L (D) and *AKAP200-S* (E) under *sev* promoter, further implying that AKAP200’s effects on Notch is unrelated to its ability to bind PKA. (F) Quantification of genotypes in (A-E) (***p = 0.0003, <0.0001 from chi square test, n = 543–746 from 3 independent eyes). (G-J) Adult wings: (G) *WT* wing. (H) Notch overexpression under *nubbin-Gal4* (expressed throughout wing) causes vein expansion and deltas, which is also observed by *AKAP200-L* (I) and *AKAP200-S* overexpression (J). (K-N) Examples of adult thoraces of indicated genotypes, red asterix indicates supernumerary bristles; (K-M) both isoforms of AKAP200 rescue its mutant phenotype (N) N-GFP,Cherry flies express an extra copy of WT Notch which also rescues the AKAP200 mutant phenotype. (O) Quantification of genotypes in (K-N) (***p<0.0001 by Mann Whitney test against *AKAP200*^*M30*^ from 10–16 flies).
(JPG)

S4 Fig AKAP200 and Notch co-localize. (A) Longer exposure of Notch co-immunoprecipitation by AKAP200-S: immunoblot from S2 cell whole cell lysates expressing Notch either in combination with Flag-control or AKAP200-S-Flag. Cell lysates were immunoprecipitated with anti-Flag antibody (IP-Flag) and blots were probed with anti-NICD antibody, revealing specific co-IP of Notch with AKAP200-S-Flag with no binding to Flag (right panel-10% input, bottom panel- blots probed with anti-Flag antibody). Upon longer exposure, there is specific binding of NEXT and NICD, but not full length Notch. (B) Schematic representation of the structure of the Notch protein. Notch receptors are type I transmembrane proteins. The extracellular domain is largely comprised of 36 EGF repeats. Egfr 8 is involved in ligand selectivity, Egfrs 11 and 12 are required for ligand binding. Following the Egfrs is the NRR, negative regulatory region, whose function is to prevent ligand independent Notch activation by concealing the S2 cleavage site. The NRR is comprised of 3 Lin-12-Notch repeats (LNR) and a hydrophobic region responsible for mediating heterodimerization (HD). The S1 and S2 cleavage sites are present here and the S3 cleavage site is present in the TM domain. NICD is composed of a RAM domain involved in CSL interaction, two NLS’s responsible for nuclear translocation, Ankyrin repeats needed for interaction with Mam, a TAD domain that recruits further coactivators needed for maximally efficient signaling, and a PEST domain targeting NICD for degradation and essential for signal termination. NEXT is comprised of the TM domain and the NICD [106]. (C) Confocal eye sections of third larval instar eye discs of *tub>AKAP200-S-Flag*, depicting localization of AKAP200-S-Flag (green), NICD (red), and E-Cad (blue, marking cellular outlines at junctional level and highlighting developing PR clusters, most strongly expressed in R2/R5, and R8, and R3/R4 also visible). Co-localization is observed between NICD punctae and AKAP200-S-Flag highlighted by white arrow (D) Confocal sections of third larval instar wing discs of *tub>AKAP200-S-Flag*, depicting the cells that give rise to the thorax. AKAP200-S-Flag (green) and NICD (red) colocalize (examples are marked by yellow arrowheads; Pearson co-efficient R = 0.4); Patj (blue) marks cellular outlines.
(JPG)

S5 Fig Quantification of Notch protein levels. (A) Western blot of third instar larval eye discs showing endogenous Notch protein; blue arrow indicates full length Notch (~290 KDa), black arrow indicates membrane bound NEXT (~120 KDa), red arrow indicates NICD (~110 KDa), all highlighted by green boxes [107]. Quantification of total Notch protein was the sum of pixel intensity of full length Notch, NEXT and NICD. See S4B Fig for schematic of Notch, highlighting the position of each Notch band/cleavage form. (B) Agarose gel electrophoresis of RNA extracted from eye disc lysate in *WT* and *AKAP200*^*M30*^ which shows no significant difference in Notch gene expression. 1 ng of RNA was used. Lanes 1,3 and 5 are RNA from eye disc lysates from *WT* flies, lanes 2,4 and 6 are RNA from eye disc lysates from *AKAP200*^*M30*^ flies. Lanes 1 and 2 were amplified with *rp49* specific primers as a positive control for the RT-PCR. Lanes 3 and 4 were amplified with *Notch* specific primers, lanes 5 and 6 used the same primers without reverse transcriptase.
(JPG)

S6 Fig AKAP200’s effect on Notch signaling is dependent on Cbl. (A) Quantification of genetic interactions shown in (B-E) of adult heads by assessing bristle number in indicated genotypes (**p = 0.001, 0.004, 0.008 from Mann Whitney tests, n = 9–24). Note, *AKAP200*^*M30*^*/+* has no phenotype- see S2A and S2F Fig. (B-E) Adult heads as representations of total bristles of indicated genotypes, loss is indicated by blue asterisks. (B) *H*^*1*^*/+* exhibits decreased bristle number. (C) Removing one genomic copy of *cbl* does not modify the *H*^*1*^*/+* phenotype. (D) Removing one genomic copy of *AKAP200*^*M3*0^ suppressed *H*^*1*^*/+*. (E) Reduced suppression of *H*^*1*^*/+* phenotype, when one genomic copy of both *AKAP200*^*M30*^ and *cbl* are removed. (F) Quantification of genetic interactions shown in (G-J) of adult nota by assessing supernumerary scutellar bristle number in indicated genotypes (**p = 0.001, from Mann Whitney tests, n = 7–35). Note *AKAP200*^*M30*^*/+* has no phenotype, see S2A and S2F Fig. (G-J) Adult scutellar bristle of indicated genotypes, red arrow indicates supernumerary bristles (G) *N*^*55e11*^*/+* shows supernumerary bristles. (H) *N*^*55e11*^*/+; cbl/+* does not vary from (G). (I) *N*^*55e11*^*/+; AKAP200*^*M30*^*/+* shows enhancement of *N*^*55e11*^*/+* phenotype. (J) *N*^*55e11*^*/+; AKAP200*^*M30*^*/+; cbl/+* does not vary from (G): when both *AKAP200* and *cbl* are reduced by one gene copy, the enhancement of the *Notch* mutant phenotype by *AKAP200* is lost. (K-L) Tangential adult eye sections of indicated genotypes. (K) PR number defects induced by *sev>N*^*Δ2155*^*/+* is not modified by the *AKAP200*^*M30*^*/+* (L). (M and N) Quantifications of the respective genotypes. (M) 1–2 are *sev>N*^*Δ2155*^*/+* and *sev>N*^*Δ2155*^*/+*, *AKAP200*^*M30*^*/+*; no significant change in phenotype is observed (chi square tests, n = 290–512 from 3–4 independent eyes). 3–5 are quantifications of genetic interaction of *sev-N*^*ΔECD*^*/+* along with the removal of one genomic copy of *AKAP200*^*M30*^ alone or both, *ago* and *AKAP200*^*M30*^. The *AKAP200*^*M30*^ mediated suppression of *sev-N*^*ΔECD*^*/+* is not affected by *ago/+* (chi square tests, n = 614–677 from 4 independent eyes). (N) Removal of one genomic copy of *Cbl* suppresses the PR number defects of *AKAP200*^*M30*^ (*** p<0.001, chi square tests, n = 387–504 from 3–4 independent eyes).
(JPG)

S7 Fig AKAP200 effect on Notch levels is dependent on lysosomal function. (A) Quantification of genetic interactions in adult nota by assessing bristle number in indicated genotypes (***p<0.0001, **p = 0.0012 from Mann Whitney’s tests, n = 11–20). (B-E) Adult heads as representations of total bristles of indicated genotypes, loss is marked by blue asterisks. (B) *H*^*1*^*/+* exhibits decreased bristle number. (C) *AKAP200*^*M30*^ suppresses the *H*^*1*^ phenotype under control condition (H_2_0 treatment) (D-E) but is unable to do so in the presence of 1 mg/ml chloroquine. Subtle deviation from the expected number of bristles (40) in WT upon exposure to 1 mg/ml of chloroquine suggests that lysosomal dysfunction has a phenotype on its own. (F) Survival rate of flies of indicated genotypes (blue and grey lines, see panel) after exposure to increasing doses of chloroquine (indicated on x-axis). (G) Confocal eye sections of third larval instar eye discs of *Ac>AKAP200-S-Flag*,UAS-*huLamp-GFP* depicting localization of AKAP200-S-Flag (red), lysosome (green), and E-Cad (blue, marking cellular outlines at junctional level and highlighting developing PR clusters, with strongest staining observed in R2/R5, and R8, and R3/R4 also visible). Minimal co-localization is observed between lysosomes and AKAP200-S-Flag. Bottom panel is a zoom of highlighted area of the top panel (R = 0.03).
(JPG)
