# Supplementary figures and images for: AKAP200 promotes Notch stability by protecting it from Cbl/lysosome-mediated degradation in Drosophila melanogaster
Source: PLoS Genet. 2018 Jan 8;14(1):e1007153. doi: 10.1371/journal.pgen.1007153 (PMC5785023; doi:10.1371/journal.pgen.1007153)

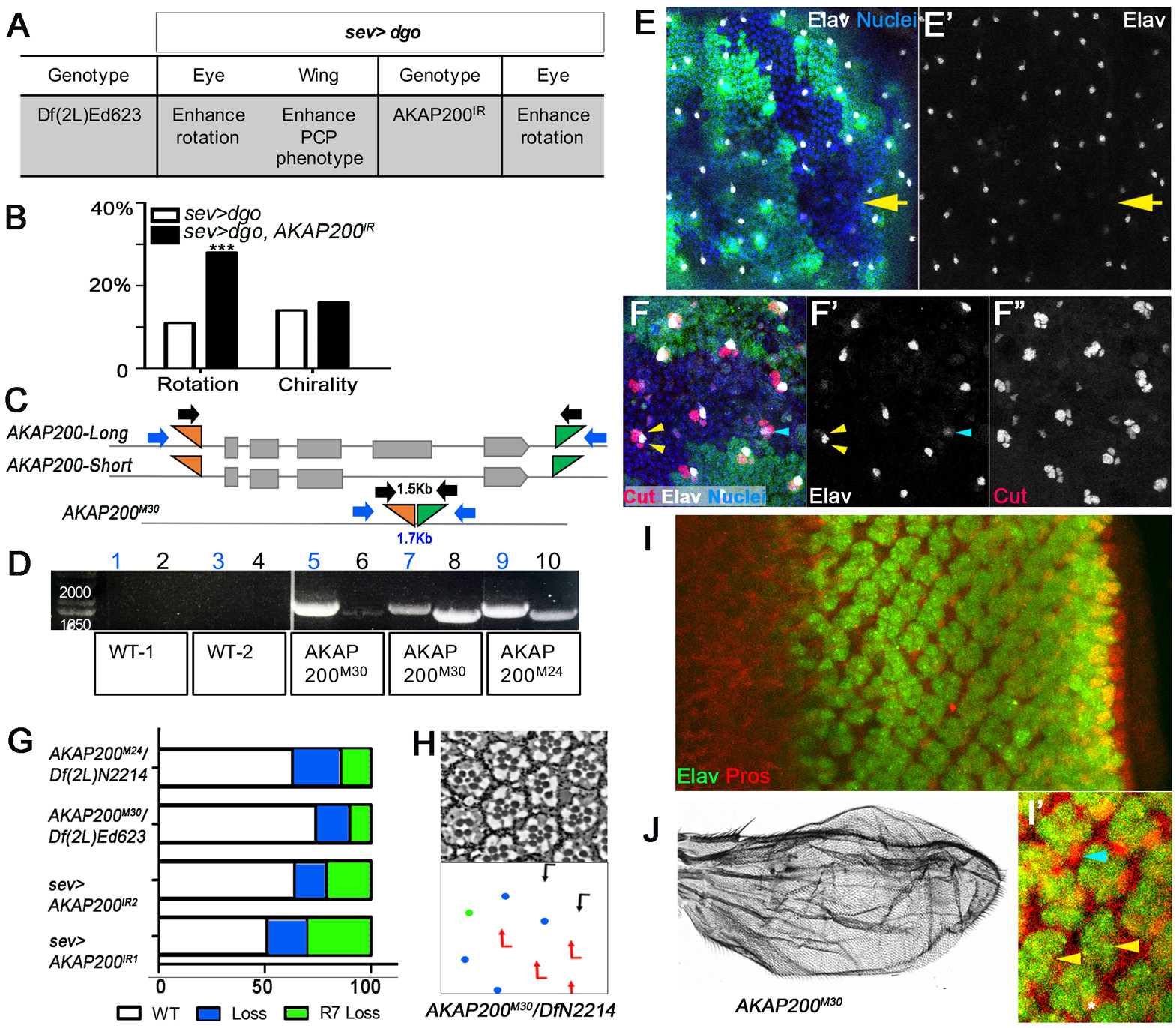

Supplement: S1 Fig — (A-B) AKAP200 was identified in a dominant modifier screen. (A) Table shown summarizes modifications of the core PCP factor dgo by AKAP200 deficiencies and IR (dgo was overexpressed by sev-Gal4 which has the sev enhancer and hs promoter leading to strong expression in R3, 4, 1, 6, 7 and cone cells, and basal expression in other tissues including the wing). (B) Quantification of genetic interactions in adult eyes: AKAP200IR enhances rotation defects from dgo overexpression (***p<0.001 from chi square test; n = 536–754, 3–4 independent eyes). (C-D) AKAP200 mutant generation and characterization: schematic of locus (C), grey bars represent coding exons of the gene. Transposable elements used to generate null mutants, P(AKAP200d03938) and Pbac(grkf07069) hereafter called XP5 and WH5 respectively, are indicated in orange and green, and flank the gene. Precise excision results in fusion of the elements and elimination of AKAP200 coding sequences. For PCR characterization, a primer pair was used that sits on elements XP5 and WH5 (depicted by black arrows), or directly outside XP5 and WH5 (depicted by blue arrows). Due to the genomic distance between the primers, in both cases, PCR amplification is only possible if the excision event happened. The expected band size for amplified band when primer combination sitting on XP5 and WH5 is used is ~1.5 Kb and for the primers outside these elements is ~1.7 Kb. (D) PCR characterization of AKAP200 null mutants from genomic DNA extracted from adult escaper flies of indicated genotypes. For lanes labeled in black, primers used sit within the transposable elements XP5 and WH5; for lanes labeled in blue, primers used sit directly outside XP5 and WH5. XP5 and WH5 are absent in WT DNA resulting in no PCR amplification, and the primers outside these elements are too far to result in PCR amplification in WT DNA. Genomic DNA from two null mutants AKAP200M30 and AKAP200M24 give a band at the expected sizes of ~1.5–1.7 Kb upon PCR amplificatio [file pgen.1007153.s001.jpg]

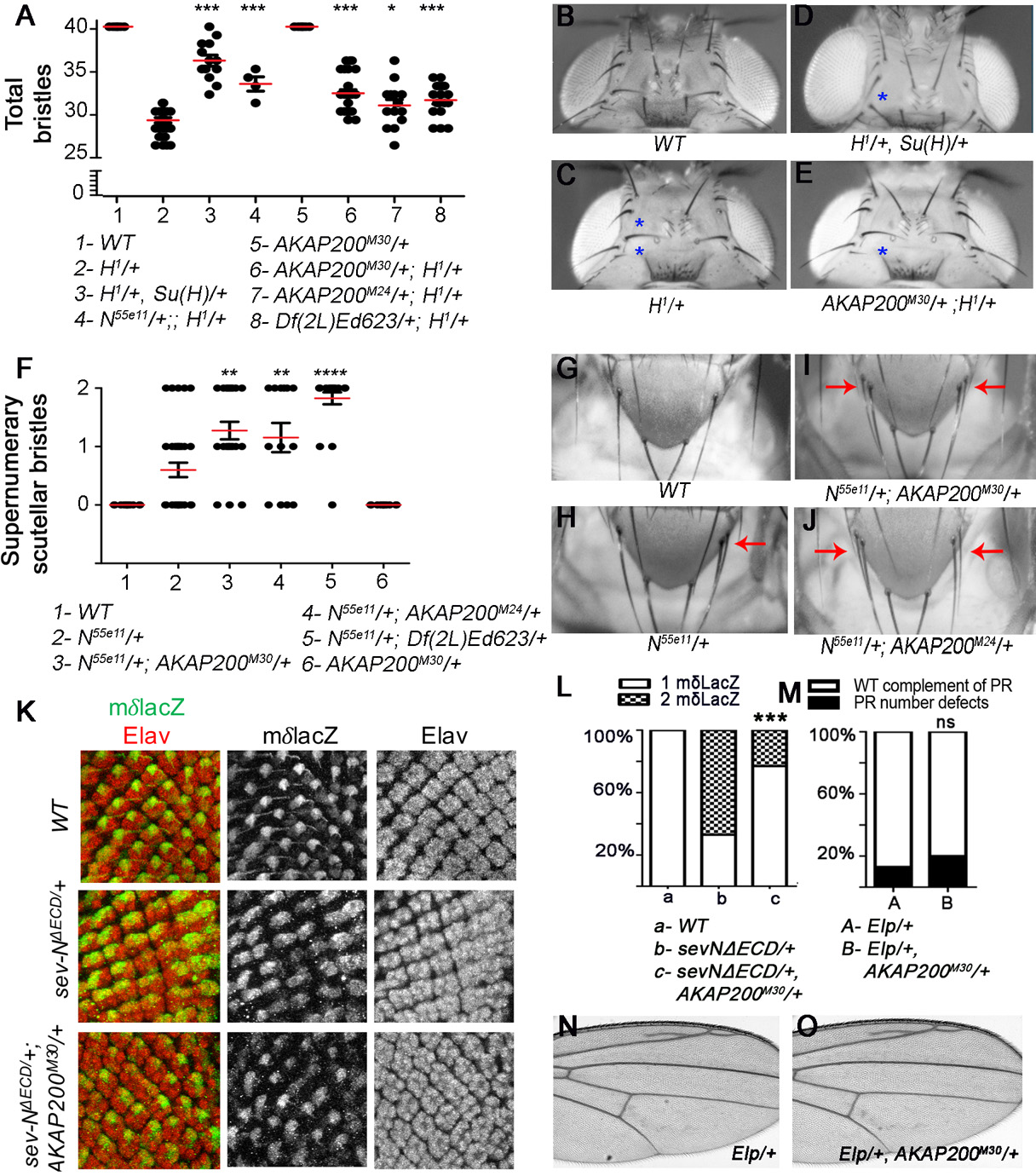

Supplement: S2 Fig — (A) Quantification of genetic interactions in adult nota by assessing bristle number in indicated genotypes. Activation of the Notch pathway using the Hairless (H) mutant, the co-repressor that keeps Notch target genes off in the absence of signal, results in decreased number of bristles compared to WT. This phenotype is dominantly rescued by removal of one copy of Su(H)[Su(H)Δ47, null allele] or Notch (N55e11, null allele). Similar suppression is observed with AKAP200 mutants (M30 and M24) and deficiency, note AKAP200M30/+ has no phenotype (***p<0.0001, *p = 0.02 by Mann Whitney test against H1/+ from 5–20 flies). (B-E) Examples of adult heads as representations of total bristles of indicated genotypes, loss of bristles is indicated by blue asterisk. (B) WT head showing normal bristle arrangement. (C) H1/+ head showing loss of bristles. (D) H1/+, Su(H)/+ and (E) AKAP200M30/+; H1/+ showing strong and moderate suppression of H1/+ loss of bristles phenotype respectively. (F) Quantification of genetic interactions in adult scutellum by assessing supernumerary bristles in indicated genotypes. Reduction of Notch signaling in N55e11 null mutant causes increase in number of bristles, which is enhanced by loss of AKAP200 mutants or deficiency, note AKAP200M30/+ has no phenotype (***p<0.0001, **p = 0.0016, *p = 0.04 by Mann Whitney test against N55e11/+ from 9–35 flies). (G-J) Examples of adult scutellar bristles of indicated genotypes, red arrow indicates supernumerary bristles. (G) WT scutellum showing normal bristle arrangement. (H) N55e11/+ showing supernumerary bristles. (I) N55e11/+; AKAP200M30/+ and (J) N55e11/+; AKAP200M24/+ show significant enhancement of N55e11/+ phenotype. (K) Confocal images of third instar eye discs of indicated genotypes stained for neuronal marker Elav (red, labeling all PR cells) and LacZ which stains mδ (Green, initially expressed at low levels in R3 and R4, following Notch activation it is upregulated in R4). One R4/ommatidium is observed i [file pgen.1007153.s002.jpg]

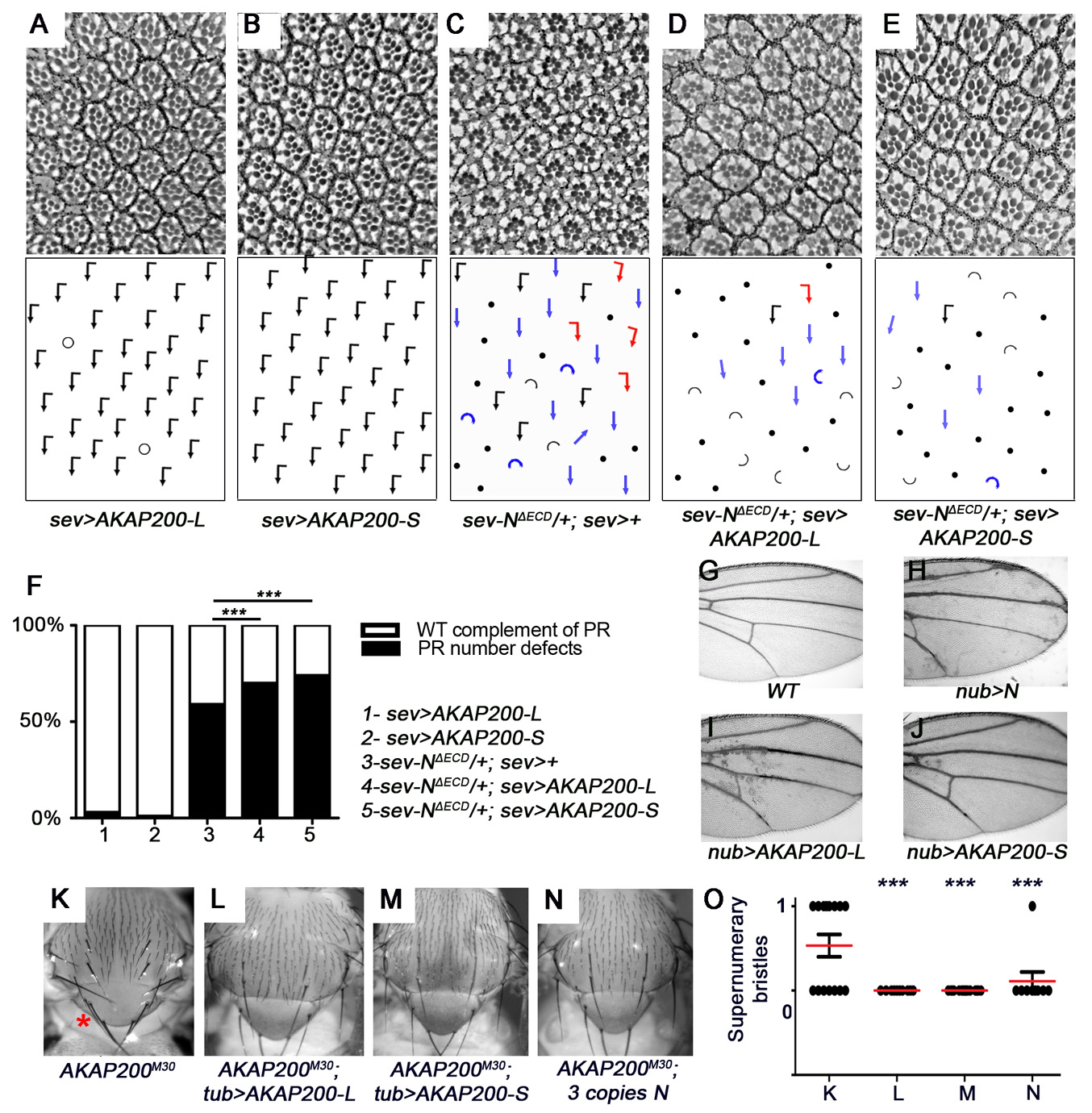

Supplement: S3 Fig — (A-E) Tangential adult eye sections of indicated genotypes (A) AKAP200-L and (B) AKAP200-S overexpression under sev-Gal4 results in <1% defects. sev-Gal4 driven overexpression produces stronger phenotypes than tub-Gal4; both isoforms of AKAP200 produced a negligible effect upon overexpression by sev-Gal4. This indicates that AKAP200 overexpression rescues the mutant with no additive effects of its own phenotype (cf. to Fig 3A). (C-E) The PR number defects induced by sev-NΔECD (C) are enhanced by co-expression of AKAP200-L (D) and AKAP200-S (E) under sev promoter, further implying that AKAP200’s effects on Notch is unrelated to its ability to bind PKA. (F) Quantification of genotypes in (A-E) (***p = 0.0003, <0.0001 from chi square test, n = 543–746 from 3 independent eyes). (G-J) Adult wings: (G) WT wing. (H) Notch overexpression under nubbin-Gal4 (expressed throughout wing) causes vein expansion and deltas, which is also observed by AKAP200-L (I) and AKAP200-S overexpression (J). (K-N) Examples of adult thoraces of indicated genotypes, red asterix indicates supernumerary bristles; (K-M) both isoforms of AKAP200 rescue its mutant phenotype (N) N-GFP,Cherry flies express an extra copy of WT Notch which also rescues the AKAP200 mutant phenotype. (O) Quantification of genotypes in (K-N) (***p<0.0001 by Mann Whitney test against AKAP200M30 from 10–16 flies). (JPG) [file pgen.1007153.s003.jpg]

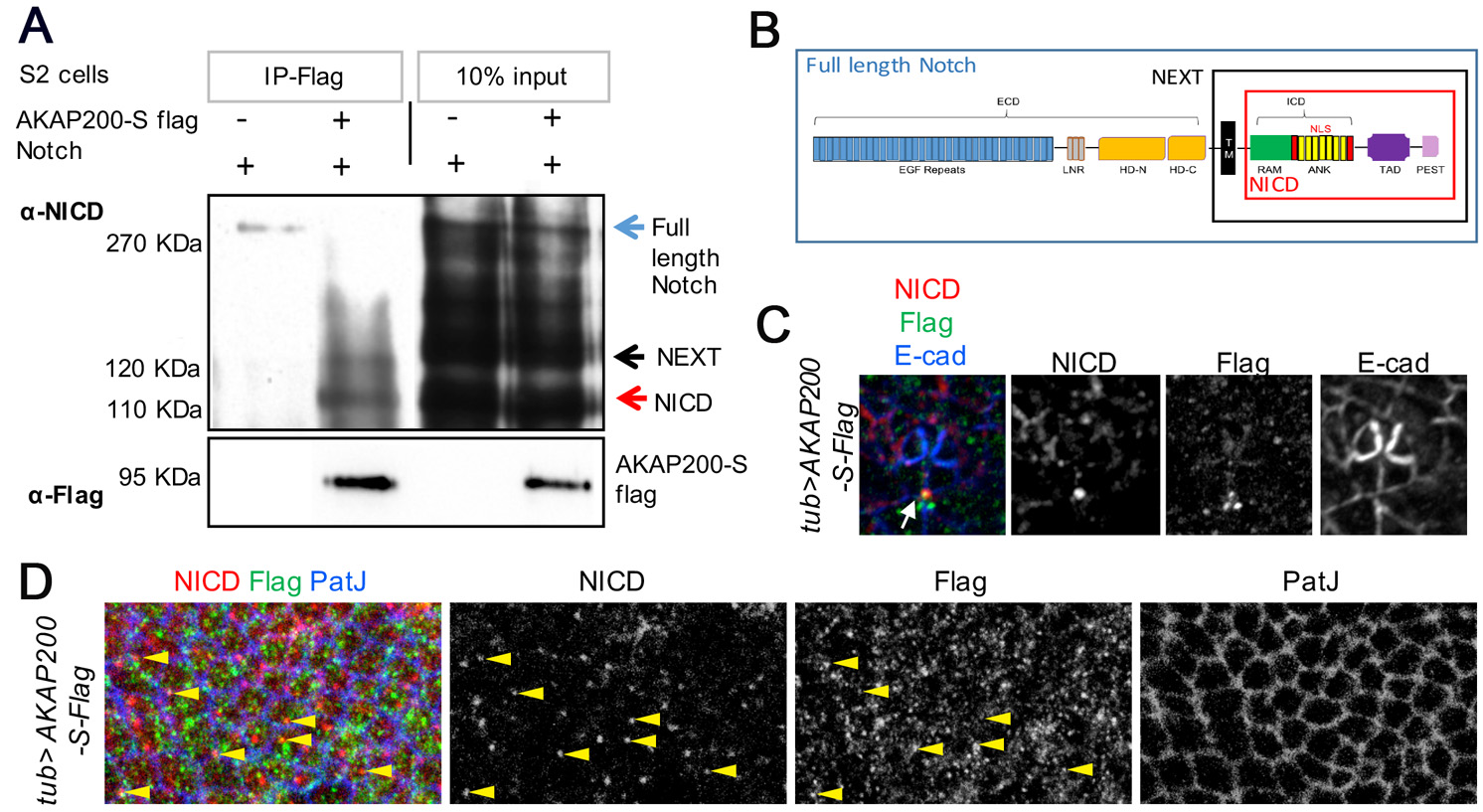

Supplement: S4 Fig — (A) Longer exposure of Notch co-immunoprecipitation by AKAP200-S: immunoblot from S2 cell whole cell lysates expressing Notch either in combination with Flag-control or AKAP200-S-Flag. Cell lysates were immunoprecipitated with anti-Flag antibody (IP-Flag) and blots were probed with anti-NICD antibody, revealing specific co-IP of Notch with AKAP200-S-Flag with no binding to Flag (right panel-10% input, bottom panel- blots probed with anti-Flag antibody). Upon longer exposure, there is specific binding of NEXT and NICD, but not full length Notch. (B) Schematic representation of the structure of the Notch protein. Notch receptors are type I transmembrane proteins. The extracellular domain is largely comprised of 36 EGF repeats. Egfr 8 is involved in ligand selectivity, Egfrs 11 and 12 are required for ligand binding. Following the Egfrs is the NRR, negative regulatory region, whose function is to prevent ligand independent Notch activation by concealing the S2 cleavage site. The NRR is comprised of 3 Lin-12-Notch repeats (LNR) and a hydrophobic region responsible for mediating heterodimerization (HD). The S1 and S2 cleavage sites are present here and the S3 cleavage site is present in the TM domain. NICD is composed of a RAM domain involved in CSL interaction, two NLS’s responsible for nuclear translocation, Ankyrin repeats needed for interaction with Mam, a TAD domain that recruits further coactivators needed for maximally efficient signaling, and a PEST domain targeting NICD for degradation and essential for signal termination. NEXT is comprised of the TM domain and the NICD [106]. (C) Confocal eye sections of third larval instar eye discs of tub>AKAP200-S-Flag, depicting localization of AKAP200-S-Flag (green), NICD (red), and E-Cad (blue, marking cellular outlines at junctional level and highlighting developing PR clusters, most strongly expressed in R2/R5, and R8, and R3/R4 also visible). Co-localization is observed between NICD punctae and AKAP200-S-Flag highlight [file pgen.1007153.s004.jpg]

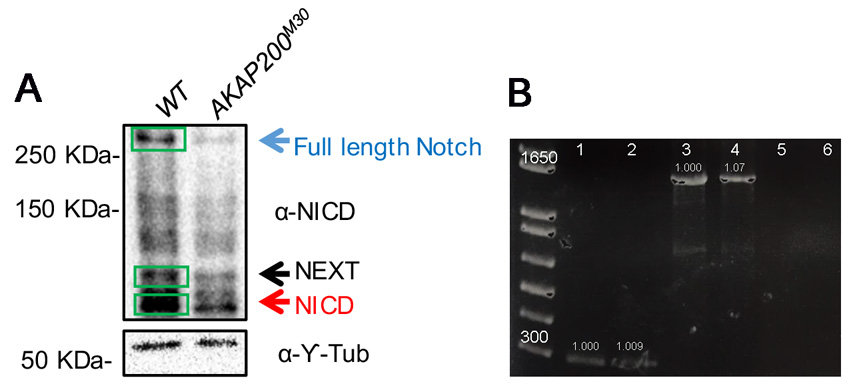

Supplement: S5 Fig — (A) Western blot of third instar larval eye discs showing endogenous Notch protein; blue arrow indicates full length Notch (~290 KDa), black arrow indicates membrane bound NEXT (~120 KDa), red arrow indicates NICD (~110 KDa), all highlighted by green boxes [107]. Quantification of total Notch protein was the sum of pixel intensity of full length Notch, NEXT and NICD. See S4B Fig for schematic of Notch, highlighting the position of each Notch band/cleavage form. (B) Agarose gel electrophoresis of RNA extracted from eye disc lysate in WT and AKAP200M30 which shows no significant difference in Notch gene expression. 1 ng of RNA was used. Lanes 1,3 and 5 are RNA from eye disc lysates from WT flies, lanes 2,4 and 6 are RNA from eye disc lysates from AKAP200M30 flies. Lanes 1 and 2 were amplified with rp49 specific primers as a positive control for the RT-PCR. Lanes 3 and 4 were amplified with Notch specific primers, lanes 5 and 6 used the same primers without reverse transcriptase. (JPG) [file pgen.1007153.s005.jpg]

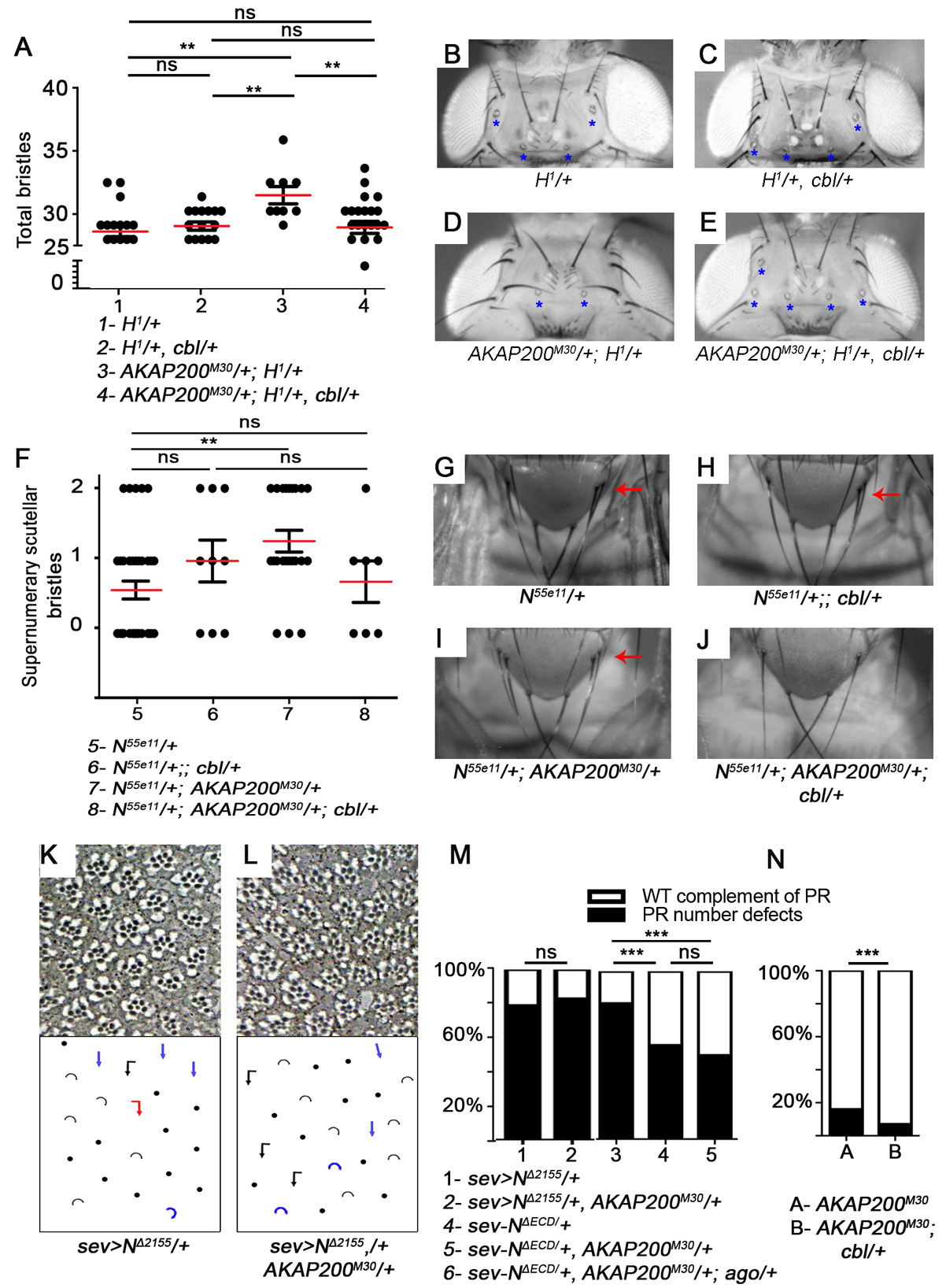

Supplement: S6 Fig — (A) Quantification of genetic interactions shown in (B-E) of adult heads by assessing bristle number in indicated genotypes (**p = 0.001, 0.004, 0.008 from Mann Whitney tests, n = 9–24). Note, AKAP200M30/+ has no phenotype- see S2A and S2F Fig. (B-E) Adult heads as representations of total bristles of indicated genotypes, loss is indicated by blue asterisks. (B) H1/+ exhibits decreased bristle number. (C) Removing one genomic copy of cbl does not modify the H1/+ phenotype. (D) Removing one genomic copy of AKAP200M30 suppressed H1/+. (E) Reduced suppression of H1/+ phenotype, when one genomic copy of both AKAP200M30 and cbl are removed. (F) Quantification of genetic interactions shown in (G-J) of adult nota by assessing supernumerary scutellar bristle number in indicated genotypes (**p = 0.001, from Mann Whitney tests, n = 7–35). Note AKAP200M30/+ has no phenotype, see S2A and S2F Fig. (G-J) Adult scutellar bristle of indicated genotypes, red arrow indicates supernumerary bristles (G) N55e11/+ shows supernumerary bristles. (H) N55e11/+; cbl/+ does not vary from (G). (I) N55e11/+; AKAP200M30/+ shows enhancement of N55e11/+ phenotype. (J) N55e11/+; AKAP200M30/+; cbl/+ does not vary from (G): when both AKAP200 and cbl are reduced by one gene copy, the enhancement of the Notch mutant phenotype by AKAP200 is lost. (K-L) Tangential adult eye sections of indicated genotypes. (K) PR number defects induced by sev>NΔ2155/+ is not modified by the AKAP200M30/+ (L). (M and N) Quantifications of the respective genotypes. (M) 1–2 are sev>NΔ2155/+ and sev>NΔ2155/+, AKAP200M30/+; no significant change in phenotype is observed (chi square tests, n = 290–512 from 3–4 independent eyes). 3–5 are quantifications of genetic interaction of sev-NΔECD/+ along with the removal of one genomic copy of AKAP200M30 alone or both, ago and AKAP200M30. The AKAP200M30 mediated suppression of sev-NΔECD/+ is not affected by ago/+ (chi square tests, n = 614–677 from 4 independent eyes). (N) Removal of one [file pgen.1007153.s006.jpg]

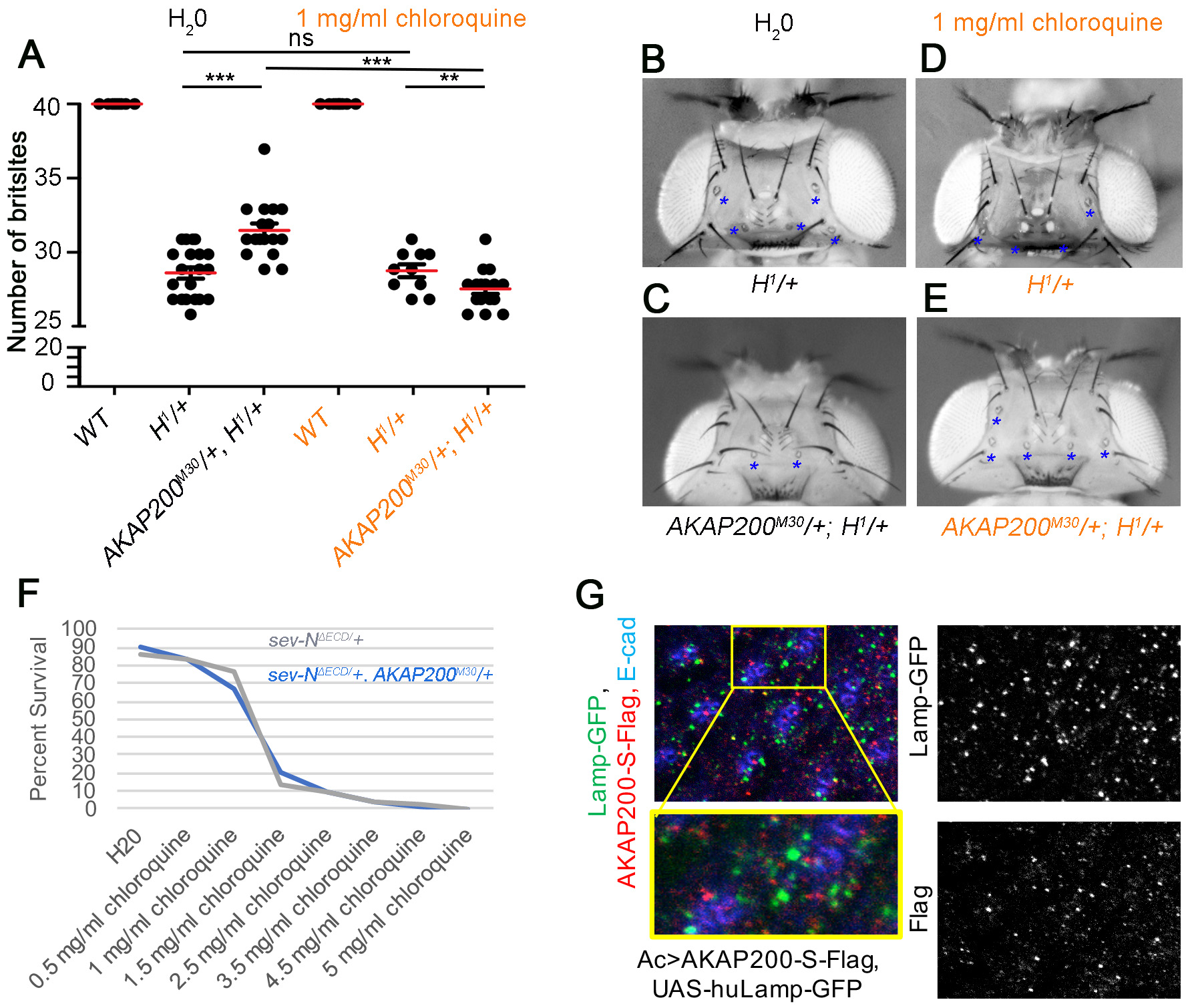

Supplement: S7 Fig — (A) Quantification of genetic interactions in adult nota by assessing bristle number in indicated genotypes (***p<0.0001, **p = 0.0012 from Mann Whitney’s tests, n = 11–20). (B-E) Adult heads as representations of total bristles of indicated genotypes, loss is marked by blue asterisks. (B) H1/+ exhibits decreased bristle number. (C) AKAP200M30 suppresses the H1 phenotype under control condition (H20 treatment) (D-E) but is unable to do so in the presence of 1 mg/ml chloroquine. Subtle deviation from the expected number of bristles (40) in WT upon exposure to 1 mg/ml of chloroquine suggests that lysosomal dysfunction has a phenotype on its own. (F) Survival rate of flies of indicated genotypes (blue and grey lines, see panel) after exposure to increasing doses of chloroquine (indicated on x-axis). (G) Confocal eye sections of third larval instar eye discs of Ac>AKAP200-S-Flag,UAS-huLamp-GFP depicting localization of AKAP200-S-Flag (red), lysosome (green), and E-Cad (blue, marking cellular outlines at junctional level and highlighting developing PR clusters, with strongest staining observed in R2/R5, and R8, and R3/R4 also visible). Minimal co-localization is observed between lysosomes and AKAP200-S-Flag. Bottom panel is a zoom of highlighted area of the top panel (R = 0.03). (JPG) [file pgen.1007153.s007.jpg]
